# Identification of Anti-Inflammatory and Anti-Proliferative Neolignanamides from *Warburgia ugandensis* Employing Multi-Target Affinity Ultrafiltration and LC-MS

**DOI:** 10.3390/ph14040313

**Published:** 2021-04-01

**Authors:** Xiao-Cui Zhuang, Yong-Li Zhang, Gui-Lin Chen, Ye Liu, Xiao-Lan Hu, Na Li, Jian-Lin Wu, Ming-Quan Guo

**Affiliations:** 1 Key Laboratory of Plant Germplasm Enhancement and Specialty Agriculture, Wuhan Botanical Garden, Chinese Academy of Sciences, Wuhan 430074, China; zhuangxiaocui@yxnu.edu.cn (X.-C.Z.); zhangyongli@wbgcas.cn (Y.-L.Z.); glchen@wbgcas.cn (G.-L.C.); liuye@wbgcas.cn (Y.L.); 2 University of Chinese Academy of Sciences, Beijing 100049, China; 3 Sino-Africa Joint Research Center, Chinese Academy of Sciences, Wuhan 430074, China; 4 Innovation Academy for Drug Discovery and Development, Chinese Academy of Sciences, Shanghai 201203, China; 5 School of Chemical Biology and Environment, Yuxi Normal University, Yuxi 653100, China; 6 State Key Laboratory of Quality Research in Chinese Medicine, Macau University of Science and Technology, Avenida Wai Long, Taipa, Macau SAR, China; 18098538ct30001@student.must.edu.mo (X.-L.H.); nli@must.edu.mo (N.L.); jlwu@must.edu.mo (J.-L.W.)

**Keywords:** *Warburgia ugandensis*, neolignanamides, affinity ultrafiltration, anti-inflammatory, anti-proliferative, cyclooxygenase-2, 5-lipoxygenase, topoisomerase I, topoisomerase II

## Abstract

Previous reports have illustrated that the incidence and mortality of cancer are increasing year by year worldwide. In addition, the occurrence, development, recurrence and metastasis of cancer are closely related to inflammation, which is a kind of defensive response of human body to various stimuli. As an important medicinal plant in Africa, *Warburgia ugandensis* has been reported to have certain anti-inflammatory and anti-proliferative activities, but its specific components and mechanisms of action remain elusive. To tackle this challenge, affinity ultrafiltration with drug targets of interest coupled to high-performance liquid chromatography-mass spectrometry (AUF-HPLC-MS/MS) could be utilized to quickly screen out bioactive constituents as ligands against target enzymes from complex extracts of this plant. AUF-HPLC-MS/MS with four drug targets, i.e., cyclooxygenase-2 (COX-2), 5-lipoxygenase (5-LOX), topoisomerase I (Top I) and topoisomerase II (Top II) were used to rapidly screen and characterize the anti-inflammatory and anti-proliferative natural ligands from *W. ugandensis*, and the resulting potential active compounds as ligands with specific binding affinity to COX-2, 5-LOX, Top I and Top II, were isolated with modern separation and purification techniques and identified with spectroscopic method like NMR, and then their antiinflammatory and anti-proliferative activities were tested to verify the screening results from AUF-HPLC-MS/MS. Compounds **1** and **2**, which screened out and identified from *W. ugandensis* showed remarkable binding affinity to COX-2, 5-LOX, Top I and Top II with AUF-HPLC-MS/MS. In addition, 1 new compound (compound **3**), together with 5 known compounds were also isolated and identified from *W. ugandensis*. The structure of compound **3** was elucidated by extensive 1D, 2D NMR data and UPLC-QTOF-MS/MS. Furthermore, compounds **1** and **2** were further proved to possess both anti-inflammatory and anti-proliferative activities which are in good agreement with the screening results using AUF-HPLC-MS/MS. This work showcased an efficient method for quickly screening out bioactive components with anti-inflammatory and anti-proliferative activity from complex medicinal plant extracts using AUF-HPLC-MS/MS with target enzymes of interest, and also demonstrated that neolignanamides (compounds **1** and **2**) from *W. ugandensis* would be the active components responsible for its anti-inflammatory and anti-proliferative activity with the potential to treat cancer and inflammation.

## 1. Introduction

*Warburgia ugandensis*, a traditional medicinal plant commonly known as pepper bark tree and greenheart in Eastern and Southern Africa, has been widely used as conventional therapeutic medicine to treat various diseases by local people for a long time [1]. Recently, many reports have illustrated the traditional uses of *W. ugandensis* in aches with pains, and inflammation, for example, stomachache, toothache, headache and joint ache, etc. [1]. Bioactivities of different solvent extracts from *W. ugandensis* were mainly manifested in anti-inflammatory [2], anti-proliferative [3], antioxidant [4], antimicrobial [5,6,7,8,9], antiparasitic activities [10,11,12], etc. However, its specific chemical components responsible for the activities above and their corresponding mechanisms of action remain need to be explored. In the last few years, there has been ever-increasing interest in its active components responsible for the anti-cancer and anti-inflammatory effects [2,3], since cancer and inflammation are becoming a growing threat to people’s health [13,14,15,16].

Up to now, cancer has become a major public devastating disease, which affected the quality of life and caused a large amount of death worldwide in the 21st century [17]. Global cancer statistics in 2018 estimated that there were 18.1 million cancer patients (approximately 53.04% died) all over the world [15]. Generally, the antineoplastic treatment foremostly includes surgical management, radiotherapy, chemotherapy and their combinations while still associated with major problems and difficult challenges such as chemoresistance and adverse effects, and natural medicines, especially targeted natural medicine tend to attract more attention because they have less toxic side effects [15,18]. Thus, more and more efforts have been made to search for natural lead compounds or drug candidates with known drug targets against cancer. Among them, DNA topoisomerase I (Top I) and DNA topoisomerase II (Top II) are well known anti-cancer drug targets highly expressed in tumor cells, and participate in the processes of DNA replication, transcription and mitosis with different structures and functions [19,20]. More strikingly, drug research endeavors with these two enzymes as important drug targets have led to a great success in the development of some effective anticancer drugs, such as camptothecin and etoposide, which have been widely used in clinic for long [21,22,23,24,25].

As for inflammation, it is an essential defense reaction to diverse internal and external stimuli factors, which is closely related to human health [26]. On the one hand, inflammation is beneficial for minimizing impending infection and injury to restore the homeostasis of tissues. On the other hand, some reports have illustrated that uncontrolled inflammation could lead to the emergence and development of some other diseases such as cancer, coronary heart disease, osteoarthritis, Alzheimer’s disease, Parkinson’s disease and so on [13,27]. In addition, the initiation and development of inflammation involve complex and coordinated function of two important proteases: cyclooxygenase-2 (COX-2) and 5-lipoxygenase (5-LOX), and several studies have confirmed that COX-2 and 5-LOX inhibitors could demonstrate crucial potential as anti-inflammatory agents [28,29,30,31,32]. Besides, inflammatory enzymes participate in regulating the processes of cancer-related immunity, inflammation, and tumorigenesis [16]. Due to the high structural diversity of natural compounds and rich medicinal plant resources, there stands a good chance to discover novel natural compounds from medicinal plants with both anti-cancer and anti-inflammatory potential which can facilitate to explain the possible mechanisms of action of some traditional herb medicine being used to treat various diseases with multiple components against multiple drug targets [17].

In order to screen for active compunds with both anti-cancer and anti-inflammatory potential from *W. ugandensis* and further explain its diversified traditional use, an integrative strategy combining enzyme affinity ultrafiltration with corresponding multiple drug targets, i.e., COX-2, 5-LOX, Top I, and Top II was developed and coupled with high-performance liquid chromatography-mass spectrometry (AUF-HPLC-MS/MS) for the rapid screening and identification of natural compounds from *W. ugandensis* with both anti-inflammatory and anti-proliferative activities. More strikingly, 2 neolignanamides were screened out and identified with remarkable binding affinity to the four drug targets of interest, i.e., COX-2, 5-LOX, Top I, and Top II enzymes, together with 4 sesquiterpenoids isolated and identified from *W. ugandensis*. It is also amazing that these 2 neolignanamides were firstly found from *W. ugandensis* and further confirmed to possess both anti-inflammatory and anti-proliferative activities by using in vitro COX-2 inhibitory assay and cell-based assays; and these findings well explained its diversified traditional usage to treat cancer and inflammation. More importantly, this study also showcased an integrative strategy combing affinity ultrafiltration using multiple target enzymes (COX-2, 5-LOX, Top I and Top II) with HPLC-MS/MS for the fast screening and identification of the potential bioactive components responsible for the corresponding activities from the crude extracts of a medicinal plant of interest. On the one hand, this strategy may lead to some important new findings concerning with the most important bioactive compounds in a highly efficient manner by saving a lot of time and money on the traditional phytochemical isolation, purification and subsequent activity tests. On the other hand, this strategy can also facilitate to explain the mechanisms of action regarding the multiple uses of a traditional medicine to treat various diseases possibly due to the unique multiple components against multiple drug targets manner as revealed in this work.

## 2. Results and Discussion

Naturally air-dried powders of stem barks from *W. ugandensis* were extracted with 95% (*v*/*v*) ethanol, and then concentrated to obtain crude extracts. Dried crude extracts were homogeneously dispersed in ultrapure water, and then successively partitioned with equal volumes of different solvents to get other four fractions for further isolation, purification, affinity ultrafiltration, and activity assays.

### 2.1. Anti-Inflammatory Activities of Different Extracts and Fractions of Stem Barks from W. ugandensis

**Figure 1:**
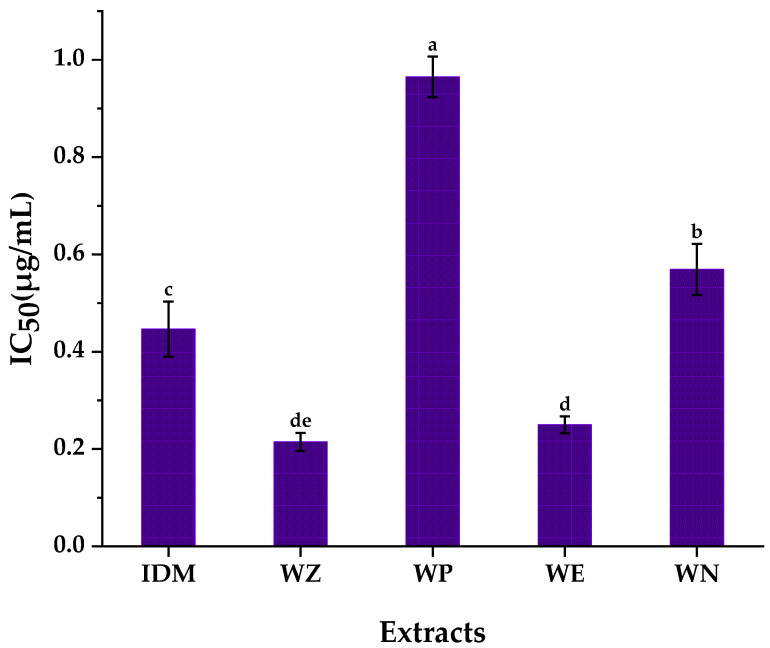
Anti-inflammatory activities of different extracts from stem barks of *W. ugandensis*. IDM: Indomethacin; WZ: 95% ethanol crude extract; WP: petroleum ether fraction; WE: ethyl acetate fraction; WN: *n*-butanol fraction; Means labelled by different letters (^a–e^) were significantly different at a level of *p* < 0.05 (n = 3) by DMRT (Duncan’s multiple range test).

COX-2 plays an important role in many physiological processes to catalyze a sequential enzyme reaction which converts arachidonic acid (AA) to prostaglandin G_2_ (PGG_2_) and prostaglandin H_2_ (PGH_2_), which have been shown to have pro-inflammatory effects [33]. Meanwhile, previous reports suggested that COX-2 can also be induced to overexpress when cells undergo inflammation and cancer, which further demonstrated that COX-2 is an essential component in tumor progression [34]. Therefore, this study started with the anti-inflammatory activities of different solvent extracts of stem barks from *W. ugandensis* to firstly explore the best effective extracts. As shown in Figure 1, 95% ethanol crude extract (WZ) and ethyl acetate fraction (WE) exhibited almost equipotent inhibitory abilities with the IC_50_ values of 0.215 ± 0.019 µg/mL and 0.249 ± 0.017 µg/mL, respectively. Both the IC_50_ values of WZ and WE were lower than indomethacin (IC_50_ = 0.446 ± 0.057 µg/mL), which was commonly used as a reference anti-inflammatory drug [35]. In comparison with this research, some studies reported sesquiterpenes which isolated from dichloromethane extract of *W. ugandensis* collected from Ethiopian, showed inhibitory effects on AA metabolites 12(*S*)-HETE and LTB4. Moreover, these sesquiterpenes also showed inhibitory capacities on 12-lipoxygenase (12-LOX) and 5-LOX [2,36]. In addition, it’s worth noting that Alfred Maroyi supposed that extracts from stem barks or leaves of *Warburgia* species for ethnomedicinal usage had anti-inflammatory activities after extensive literature research [37]. Indeed, this study similarly suggested that WZ and WE from stem barks of *W. ugandensis* displayed good anti-inflammatory activities. Considering the reduced complexity from WZ to WE, we selected one portion of WE (hereafter we simplified it as WE-P) for further evaluation aiming to explore the major specific compounds responsible for the remarkable anti-inflammatory activity of WE-P, which remain unexplored to date.

### 2.2. AUF-HPLC-MS/MS Analysis of COX-2/5-LOX/Top I/Top II Ligands

#### 2.2.1. Screening for the Potential COX-2/5-LOX/Top I/Top II Ligands in WE-P

Recently, affinity ultrafiltration was based on the interactions between small bioactive molecules and their correlative target enzymes, and has been widely used for rapidly screening out bioactive components [26,32,38]. Generally, peak area ratios of components which exhibited higher binding affinity capacities with target enzymes would be significantly different from that of lower or no binding abilities upon affinity ultrafiltration [39]. Whereupon, AUF-HPLC-MS/MS could help to rapidly screen and identify the bioactive compounds from complex plant extracts by comparing the variations of peak areas before and after ultrafiltration [40].

**Figure 2:**
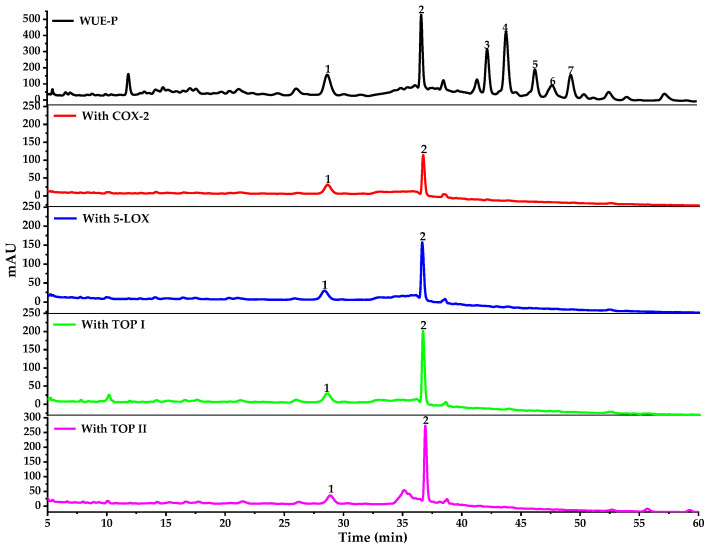
The HPLC-UV chromatograms of components in WE-P from *W. ugandensis* by ultrafiltration. The black line represents HPLC-UV profiles of WE-P from *W. ugandensis* without ultrafiltration, the red line represents HPLC-UV profiles of WE-P from *W. ugandensis* with COX-2, the blue line represents HPLC-UV profiles of WE-P from *W. ugandensis* with 5-LOX, the green line represents HPLC-UV profiles of WE-P from *W. ugandensis* with Top I, the purple line represents HPLC-UV profiles of WE-P from *W. ugandensis* with Top II, respectively.

Potential bioactive components from WE-P of *W. ugandensis* were directly analyzed by HPLC-UV/ESI-MS/MS after incubation with COX-2, 5-LOX, Top I or Top II, and then affinity ultrafiltration. As shown in Figure 2, it’s easy to recognize compounds exhibiting specific bindings to COX-2, 5-LOX, Top I or Top II from WE-P of *W. ugandensis* according to the HPLC chromatograms. Obviously, peaks 1 and 2 showed apparent peaks after ultrafiltration with those 4 activated enzymes, while peaks 3–7 showed almost no peaks. Accordingly, it could be preliminarily speculated that peaks 1 and 2 showed relative higher potent binding capacities with COX-2, 5-LOX, Top I and Top II compared with other peaks (peaks 3–7).

The relative binding strength (RBS) was employed to compare the variation of the correlated peak areas in the AUF-HPLC-UV chromatograms before and after incubation with enzymes to evaluate the affinity binding abilities between potential ligands and target enzymes [29]. The RBS formula is expressed as: RBS = (A_active_/A_sample_) × 100%, where the A_active_ represents the peak areas obtained from the WE-P samples incubated with COX-2, 5-LOX, Top I or Top II, respectively; the A_sample_ represents the peak areas obtained from WE-P samples without ultrafiltration. Table 1 showed that the RBS of peak 2 with COX-2, 5-LOX, Top I and Top II were higher than that of peak 1. Namely, the special binding capacities of peak 2 with COX-2, 5-LOX, Top I and Top II might be stronger than that of peak 1. In addition, both peaks 1 and 2 displayed the highest binding affinity with Top I followed by Top II among other peaks as shown in Table 1. Therefore, it could be preliminarily estimated that peak 1 and peak 2 were the main bioactive components in WE-P which were closely related to its anti-inflammatory and anti-proliferative activities. These compounds were then identified based on their MS/MS data, related references and NMR or UPLC-QTOF-MS/MS data of isolated compounds, and the results are shown in Table 2.

#### 2.2.2. Structural Identification of the Potential COX-2/5-LOX/Top I/Top II Ligands

Peak 1 and peak 2 from WE-P of *W. ugandensis*, which were obviously different from other 5 peaks (peaks 3–7), exhibited specific binding capacities to COX-2, 5-LOX, Top I and Top II after affinity ultrafiltration screening as shown in Figure 2 and Table 1. Table 2 listed the HPLC-MS/MS data of components from WE-P of *W. ugandensis* which included retention time (Rt), quasi-molecular ions ([M-H]^−^) and characteristic MS/MS fragments in the negative mode, and the UPLC-QTOF-MS/MS data of isolated compounds which included quasi-molecular ions ([M+H]^+^ or [M-H]^−^) and characteristic MS/MS fragments in the positive or negative mode. Theoretically, these components detected could be identified according to the AUF-HPLC-MS/MS, UPLC-QTOF-MS/MS and reported literatures. As a result, peaks 1–2 and peaks 3–6 were successively identified by comparing their retention time, their corresponding molecular ion peaks and MS/MS fragments shown in Table 2.

Compared with corresponding molecular ion peaks and MS/MS fragments of isolated compounds, peak 1 (*m*/*z* 623, [M–H]^−^), peak 2 (*m*/*z* 623, [M–H]^−^), peak 4 (*m*/*z* 265, [M–H]^−^), peak 5 (*m*/*z* 323, [M–H]^−^), and peak 6 (*m*/*z* 237, [M–H]^−^) were characterized as *N*-*cis*-grossamide, *N*-*trans*-grossamide, ugandenial A, 11*α*-hydroxycinnamosmolide and polygonal acid [41,42,43,44], respectively. Peak 1 and peak 2, belonged to neolignanamides, showed almost same cross-linked *N*-*cis*/*trans*-feruloyltyramine dimer skeletons and determined as a pair of geometrical *cis*-*trans* isomers by NMR data of isolated compound **1** and compound **2**, respectively. Cross-linked *N*-*cis*/*trans*-feruloyltyramine dimer skeleton ion of compound **1** and compound **2** (*m*/*z* 625, [M+H]^+^) produced a series of daughter ions of *m*/*z* 488 [M-tyramine+H]^+^, *m*/*z* 351 [M-2tyramine+H]^+^, and *m*/*z* 307 [M-2tyramine-CO_2_+H]^+^, which play an important role in structural identification, have been reported in previous study [42]. In this same way, peaks 4-6 were identified as sesquiterpenoids by comparing MS/MS fragments of isolated compounds in UPLC-QTOF-MS/MS with MS/MS fragments of components in HPLC-MS/MS. Particularly, peak 5 showed a skeleton of sesquiterpenoid with a series of daughter ions of *m/z* 279 [M–CO_2_–H]^−^, *m/z* 263 [M–C_2_H_4_O_2_–H]^−^, *m/z* 235 [M–C_3_H_4_O_3_–H]^−^, *m/z* 219 [M–C_4_H_7_O_3_–H]^−^, and *m/z* 191 [M–C_5_H_7_O_4_–H]^−^, and considered to be 11*α*-hydroxycinnamosmolide compared with UPLC-QTOF-MS-MS fragments of isolated compound **5** [45]. Similarly, peak 4 shared almost same fragmentation method with peak 5 by losing CO_2_ and H_2_O, determined as ugandenial A [3]. As mentioned above, the quasi-molecular ion of peak 6 (*m/z* 237 [M–H]^−^) produced a series of daughter ions of *m/z* 191 [M–H_2_O–CO–H]^−^, and *m/z* 163 [M–H_2_O–2CO–H]^−^. Therefore, peak 6 was considered to be polygonal acid by comparing the NMR data of compound **6** with previous study [41].

### 2.3. Compounds Isolated and Identified from WE-P

**Figure 3:**
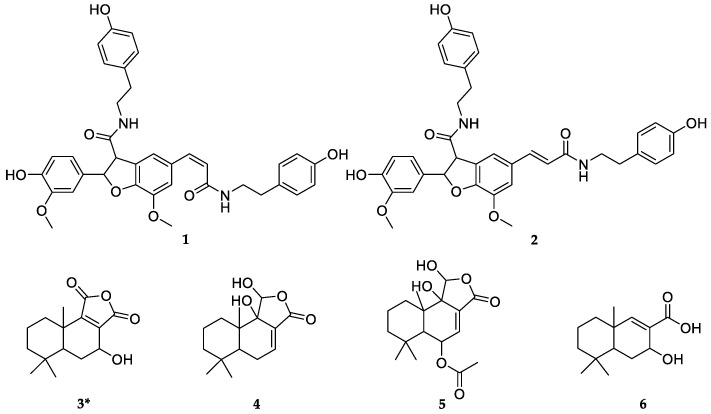
Compounds isolated and identified from WE-P; *: New compound.

Theoretically, bioactive components in WE-P could be tentatively identified by characteristic MS/MS fragments as compared to known databases, standards or isolated compounds. For some unknowns or new compounds, the isolation of pure compounds would be beneficial to subsequent structure elucidation and activity verification. In this context, compounds **1**–**6** were isolated from WE-P by using modern separation and purification techniques, including column chromatography (CC), thin layer chromatography (TLC) and HPLC guided by the HPLC-MS/MS. Their structures were established by NMR (Figures S1–S15) and UPLC-QTOF-MS/MS as follows: *N*-*cis*-grossamide (1) [42], *N*-*trans*-grossamide (2) [42], 7-hydroxywinterin (3) [46], ugandenial A (4) [3], 11*α*-hydroxycinnamosmolide (5) [43], and polygonal acid (6) [47]. Among them, compounds **1** and **2**, two neolignanamides, were reported firstly in *W. ugandensis*, and compound **3** was isolated as a new sesquiterpenoid as shown in Figure 3.

**Figure 4:**
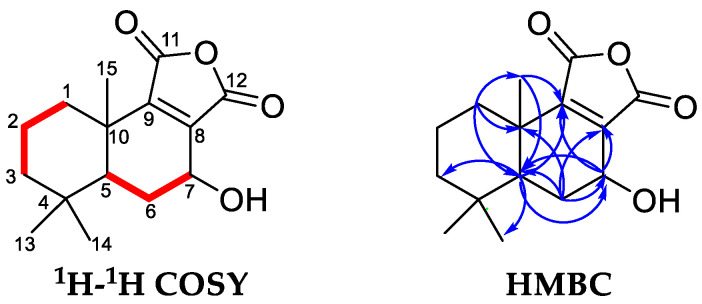
Key ^1^H-^1^H COSY and HMBC correlations for compound **3**.

Compound **3**, UV (MeOH) *λ*_max_ (log ε) 205 (3.57), was obtained as white crystalline solid. Its molecular formula was tentatively established as C_15_H_20_O_4_ on the basis of UPLC-QTOF-MS/MS analysis in the negative ion mode, with a [M–H]^−^ peak observed at *m*/*z* 263.1284 (calcd for C_15_H_20_O_4_, 263.1289), suggesting 6 degrees of unsaturation. The presence of daughter ions *m*/*z* 235 [M–CO–H]^−^, *m*/*z* 219 [M–CO_2_–H]^−^, and *m*/*z* 191 [M–CO_2_–CO–H]^−^ could be speculated that compound **3** possibly shared similar sesquiterpenoid skeleton with compounds **4** and **5**. The ^1^H NMR spectrum of compound **3** (Table 3) indicated the presence of three quaternary methyls (*δ*_H_ 0.94, 0.96 and 1.18, each singlet), and one oxymethine proton (*δ*_H_ 4.53, H-7). The ^13^C NMR and DEPT resonances were identified as three methyls (*δ*_C_ 19.1, 21.9 and 33.6), four methylenes (*δ*_C_ 19.4, 29.4, 35.8 and 42.7), two methines (*δ*_C_ 47.0 and 60.3), four quaternary carbons (*δ*_C_ 33.8, 38.1, 139.8, and 153.9), two carbonyl carbons (*δ*_C_ 172.3 and 172.7). Accordingly, this compound could be ascribed to a drimane sesquiterpenoid skeleton [3,48,49,50,51]. The ^1^H-^1^H COSY and HMBC spectrum (Figure 4) were used to determine the structure of compound **3** (Figure 3). On the one hand, interpretation of the ^1^H-^1^H COSY data revealed the correlations of H-1/H-2, H-2/H-3, H-5/H-6, and H-6/H-7, which confirmed the existence of two structure fragments: –CH_2_–CH_2_–CH_2_– and –CH–CH_2_–CH–O- as shown in Figure 4. On the other hand, the HMBC spectrum showed H-1 was correlated with C-5, C-10, and C-15; H-5 was correlated with C-3, C-7, C-9, and C-14; H-6 was correlated with C-5, C-7, C-8, and C-10; H-7 was correlated with C-5, C-8, and C-9; H-15 was correlated with C-5 and C-9. Among them, the correlations related to H-7 and C-7 in the HMBC data suggested that a hydroxy group was placed at C-7. Thus, compound **3** was identified as a new derivative of winterin [46], namely the 7-hydroxywinterin.

### 2.4. Anti-Inflammatory Activities of Compounds Isolated and Identified from WE-P

**Figure 5:**
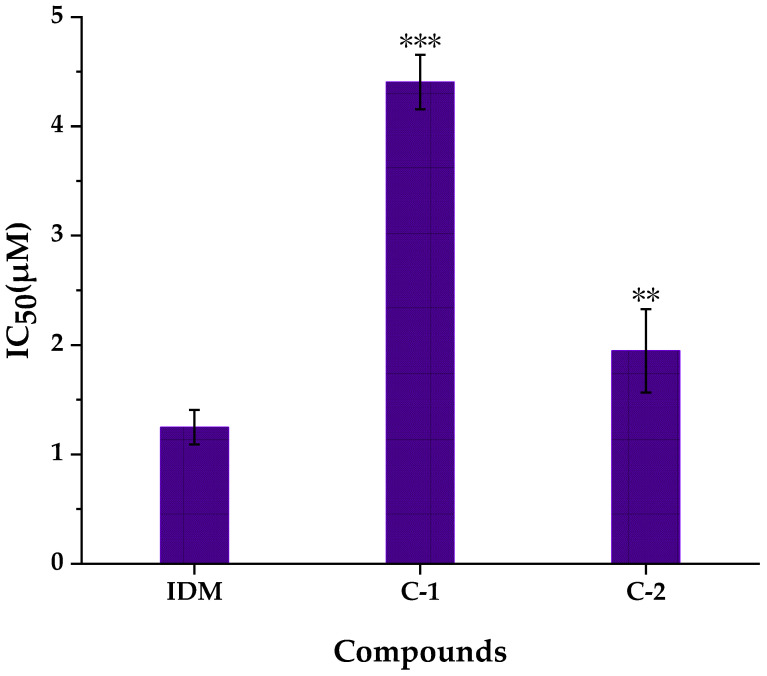
COX-2 inhibitory activities of compounds isolated from WE-P. IDM, Indomethacin; C-1, compound **1**; C-2, compound **2**; IC_50_: The half-maximal inhibitory concentrations. ** *p* < 0.01 and *** *p* < 0.001, compared with indomethacin group (IDM).

The conversions of arachidonic acid (AA) to prostaglandin G_2_ (PGG_2_), and PGG_2_ to prostaglandin H_2_ (PGH_2_) were catalyzed by inducible enzyme COX-2 [29,33]. COX-2 is the main targets of non-steroidal anti-inflammatory drugs (NSAIDs), it is induced to overexpress especially during the inflammatory and neoplastic processes [33]. As shown in Figure 5, compounds **1** and **2** (geometric isomerism of compound **1**) exerted inhibitory activities on COX-2 with the IC_50_ values of 4.405 ± 0.249 µM and 1.948 ± 0.381 µM, respectively. That is to say, the *N*-*trans*-grossamide (compound **2**) exhibited better anti-inflammatory activities than the *N*-*cis*-grossamide (compound **1**). This result was consistent with the reported literature [52]. As reported in previous study, compound **2** was evaluated to show substantially more potent anti-inflammatory capacities than the positive standard ibuprofen by measuring the levels of 4 kinds of cytokines, interleukin-1*β* (IL-1*β*), interleukin-2 (IL-2), granulocyte-macrophage colony stimulating factor (GM-CSF) and tumor necrosis factor-*α* (TNF-α) in the supernatant media of human peripheral blood mononuclear cells (PBMCs) stimulated by lipopolysaccharide (LPS) at the tested concentration of 100 µM. As a result, compound **2** decreased the release of IL-1*β* (2.14% of LPS control), IL-2 (11.61% of LPS control), GM-CSF (1.61% of LPS control), TNF-α (0.06% of LPS control) [53]. Furthermore, compound **2** showed moderate inhibition of nitric oxide (NO) production stimulated by LPS in the RAW 264.7 cell line with IC_50_ value of 52.5 ± 6.1 µM in previous study [52]. In conclusion, the inhibitory activities of compound **1** and **2** on COX-2 were correlated with the results of affinity ultrafiltration screening (in Section 2.2.), which speculated that compound **1** and compound **2** were potent ligands for COX-2 and 5-LOX. In our previous reports on *W. ugandensis*, lignanamides and phenolic amides were determined to show good inhibitory activities against COX-2 [54]. Therefore, neolignanamides (compounds **1** and **2**), like lignanamides and phenolic amides, would be characteristic components of *W. ugandensis* to exert potential anti-inflammatory activities.

### 2.5. Anti-Proliferative Activities of Compounds Isolated and Identified from WE-P

**Figure 6:**
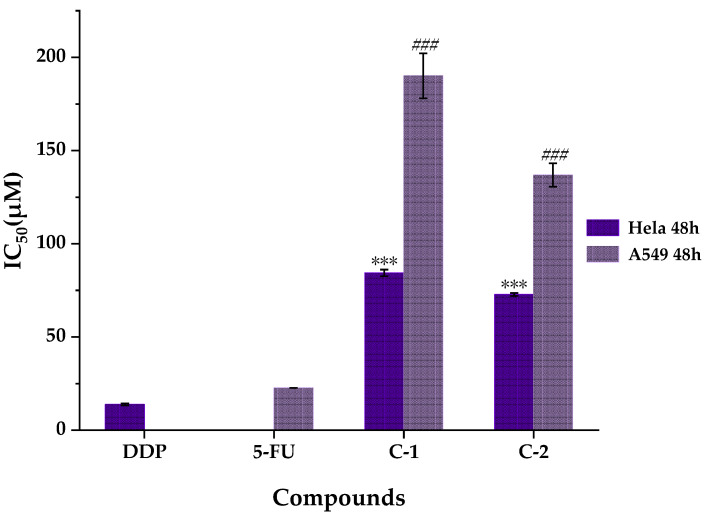
Anti-proliferative activities of compounds isolated from WE-P of *W. ugandensis*. DDP, Cisplatin; 5-FU: 5-Fluorouracil; C-1, compound **1**; C-2, compound **2**; Hela: Human cervical cancer cell line; A549: Human non-small cell lung adenocarcinoma cell line; IC_50_: The half-maximal inhibitory concentrations. *** *p* < 0.001, compared with cisplatin group (DDP); *^###^ p* < 0.001, compared with 5-fluorouracial group (5-FU).

Nowadays, lung cancer is a prevalent malignancy which has the highest incidence and death rate all over the world [18]; cervical cancer becomes the fourth leading cause of cancer-related death in women [14]. It has been reported that *W. ugandensis* showed good anticancer activity on KB cell line [3]. To explore the potential responsible compounds from it, the anti-proliferative activities of compounds isolated and identified from WE-P were further evaluated using Hela cells and A549 cells treated with various concentrations of compounds with the indicated time points, and the cell viabilities were measured via SRB assay. As shown in Figure 6, for the Hela cells, compound **1** and compound **2** exhibited moderate anti-proliferative activities with the IC_50_ values of 84.360 ± 1.794 µM and 72.803 ± 0.839 µM, respectively. In addition, the inhibitory activities of compound **1** and **2** were consistent with the results of affinity ultrafiltration screening (in Section 2.2.), which suggested that compound **1** and compound **2** were potential ligands for Top I and Top II. Meanwhile, previous study supposed that compound **2** displayed promising anti-proliferative activities against Hela cells and human mammary adenocarcinoma (MCF-7) cells with IC_50_ values of 20.10 ± 3.05 µM and 11.43 ± 1.62 µM, respectively [55]. Similarly, compound **2** exhibited moderate anti-proliferative activity on Hela cell line [56], and also displayed moderate cytotoxicity in cultured LNCaP human prostate cancer cell [57], which confirmed the anti-proliferative capacities of compound **2**. In addition, compound **2** was also suggested to be moderately active against MCF-7 and human prostate cancer (PC-3) cells, with GI_50_ (the 50% growth inhibition in 72 h) values of 24.0 and 21.0 µM, respectively [58]. As for the A549 cells, both the IC_50_ values of compounds **1** and **2** were more than 100 µM. In a word, neolignanamides **1** and **2** showed relatively worse anti-proliferative activities on A549 cells than Hela cells. Differently, previous study suggested that compound **2** displayed promising anti-proliferative activities against A549 cells with IC_50_ value of 13.53 ± 3.66 µM [55]. As shown in previous report, compound **2** was indicated to be inactive or poorly active (GI_50_ > 100 µM) against human colorectal adenocarcinoma (Caco-2) cells. Besides, derivatives of dihydrobenzofuran neolignanamides which were acquired from biomimetic synthesis with minimal structural diversification with respect to compound **2**, were suggested to show potent anti-proliferative activities against Caco-2, MCF-7 and PC-3 [58]. Based on the results mentioned above, neolignanamides **1** and **2** can be used as anti-inflammatory and anti-proliferative lead compounds for biomimetic synthesis or synthesis in order to quickly discover more potent anti-inflammatory and anti-proliferative agents in the near future.

## 3. Materials and Methods

### 3.1. Plant Material

Stem barks of *W. ugandensis* were collected by Professor Mingquan Guo in June 2018 from Narok County, Kenya, and authenticated by taxonomist Guangwan Hu. A voucher specimen (No. WBG-ZWHX 201808001) was deposited in the herbarium of the Key Laboratory of Plant Germplasm Enhancement and Specialty Agriculture, Wuhan Botanical Garden, Chinese Academy of Sciences.

### 3.2. Chemicals and Reagents

Cisplatin (DDP), 5-fluorouracil (5-FU), and indomethacin (IDM) were purchased from Sigma-Aldrich Corp. (Shanghai, China). COX-2 (human) inhibitor screening assay kit (No. S0168) was obtained from Beyotime Biotechnology (Shanghai, China). COX-2 was purchased from Wuhan Antgene Biotechnology Co., Ltd. (Wuhan, China). 5-LOX was purchased from Cayman Chemical Co., Ltd. (Ann Arbor, MI, USA). Top I and Top II were purchased from New England Biolabs (Ipswich, USA). Millipore membranes (Φ 13 mm, 0.22 µm) were provided by Tianjin Jinteng Experiment Equipment Co., Ltd. (Tianjin, China). Ten kDa (YM-10) ultrafiltration membranes were purchased from Millipore Co. Ltd. (Bedford, MA, USA). Ultrapure water was produced by the ultrapure water polishing system produced by Nanjing Yeap Esselte Technology Development Co., Ltd. (Nanjing, China). Analytically pure chemicals and solvents were purchased from Sinopharm Chemical Reagent Co., Ltd. (Shanghai, China), for example, acetonitrile (ACN), methanol (MeOH), ethanol (EtOH), dimethyl sulfoxide (DMSO), petroleum ether (PE), ethyl acetate (EA), *N*-butanol (NBA), formic acid (FA), concentrated sulfuric acid (H_2_SO_4_), sodium hydroxide (NaOH), tris(hydroxymethyl)aminomethane (Tris), hydrochloric acid (HCl), ferric chloride (FeCl_3_) and potassium ferricyanide (K_3_[Fe(CN)_6_]). Chromatographically pure ACN, MeOH, iso-propyl alcohol (IPA) and n-hexane (NHA) were purchased from Tedia Company Int., (Fairland, OH, USA).

### 3.3. Instruments and Materials

The HPLC-UV/ESI-MS/MS was performed by Thermo Accela 600 series HPLC tandem TSQ Quantum Access MAX mass spectrometer (Thermo Fisher Scientific, San Jose, CA, USA) with a Waters Symmetry RP-C18 column (4.6 mm × 250 mm, 5 µm). UV absorbance was tested by multifunctional microplate reader (Tecan Infinite M200 PRO, TECAN, Männedorf, Switzerland). UPLC-QTOF-MS/MS data were collected by ultra-performance liquid chromatography quadrupole time of flight mass spectrometry (Agilent Technologies, Santa Clara, CA, USA) with an ACQUITY UPLC BEH column (50 × 2.1 mm, 1.7 µm), (ACQ, Waters Co., Milford, MA, USA). NMR spectra were tested on a Bruker Avance III 600 MHz (Bruker, Karlsruhe, Germany). CC was undertaken on AB-8 macroporous adsorbent resin (Qingdao Haiyang Chemical Co., Ltd., Qingdao, China), ODS-A-HG (12 nm, S-50 µm, YMC Co., Ltd., Tokyo, Japan), and Sephadex LH-20 (25–100 µm, Pharmacia Fine Chemical Co., Ltd., Uppsala, Sweden). Analytic HPLC was carried out using an Agilent 1220 liquid chromatograph (Agilent Technologies, Santa Clara, CA, USA) with a Waters Symmetry RP-C18 column (4.6 mm × 250 mm, 5 µm). Semipreparative HPLC was carried out using an Agilent 1100 liquid chromatograph (Agilent Technologies, Santa Clara, CA, USA) and a Hanbon NS4205 chromatograph (Hanbon Sci. & Tech., Jiangsu, China), with the columns of a YMC-Pack ODS-A (250 mm × 9.4 mm, 5 µm) (YMC Co., Ltd., Tokyo, Japan) and an Agilent Eclipse XDB-C18 (250 mm × 9.4 mm, 5 µm) (Agilent Technologies, Santa Clara, CA, USA). Preparative HPLC was carried out using a Hanbon NS4205 chromatograph (Hanbon Sci. & Tech., Jiangsu, China), with the column of Smuwei C18 (250 mm × 30 mm, 10 µm) (Hanbon Sci. & Tech., Jiangsu, China). TLC (Qingdao Haiyang Chemical Co., Ltd., Qingdao, China) was used directly for quick separation.

### 3.4. Preparation of Samples

#### 3.4.1. Preparation of Extracts and Fractions

Naturally dried stem barks of *W. ugandensis* (4.8 kg) were crushed with a grinder to obtain relative uniform powder, extracted with 95% (*v*/*v*) ethanol (30 L every time, 3 times) for 3 times at room temperature for 48 h every time, and then concentrated to obtain WZ (391.4 g). WZ were homogeneously dispersed in ultrapure water (1 L), and then successively extracted with equal volumes of PE, EA and NBA for 3 times to get 5 fractions: PE fraction (WP), EA fraction (WE), NBA fraction (WN) and H_2_O fraction (WH), respectively. After that, WE (91.1 g) was separated by macroporous adsorbent resin (AB-8) CC using gradient elution with EtOH-H_2_O (25–95%, *v*/*v*). And the 60% EtOH-H_2_O eluted fraction was named as WE-P for further research.

#### 3.4.2. Isolation of Compounds from WE-P

WE-P (8.1 g) was separated by reverse phase silica gel (ODS-A-HG) CC using gradient elution with gradient of MeOH in H_2_O from 38% (*v*/*v*) to 68% (*v*/*v*) to produce 6 subfractions: P1-P6. Subfraction P2 (1.5 g) was subjected to Sephadex LH-20 CC and eluted with MeOH to get 4 subfractions: P21-P24. Subfraction P22 (420.7 mg) was separated by preparative HPLC with 26% ACN-H_2_O (flow rate 2.5 mL/min) to obtain 9 subfractions: P221-P229. Subfraction P223 (51.4 mg) was purified by semipreparative HPLC with 18% ACN-H_2_O (H_2_O contains 0.1% FA, flow rate 2.5 mL/min) to yield compound **1** (1.2 mg, *R*t = 16 min), compound **2** (3.5mg, Rt = 22 min) and compound **4** (3.5mg, *R*t = 36 min). Subfraction P3 (1.2 g) was subjected to Sephadex LH-20 CC and eluted with MeOH to get 4 subfractions: P31-P34. Subfraction P32 (508.3 mg) was separated by preparative HPLC with 70% MeOH-H_2_O (flow rate 2.5 mL/min) to obtain 3 subfractions: P321-P323. Subfraction P322 (215.4 mg) was purified by semipreparative HPLC with 64% MeOH-H_2_O (flow rate 2.5 mL/min) to yield compound **5** (79.1 mg, Rt = 41 min), compound **3** (2.0 mg, *R*t = 59 min), and compound **6** (17.5 mg, *R*t = 100 min).

### 3.5. Screening and Identification of the Potential Ligands with AUF-HPLC-MS/MS

#### 3.5.1. Affinity Ultrafiltration Procedures

Affinity ultrafiltration screening was slightly modified and carried out according to some previous studies [26,32,38]. Firstly, approximately 100 µL of WE-P (10.0 mg/mL) were incubated with 10 µL of enzymes (2 U, COX-2, 5-LOX, Top I or Top II), and 90 µL of Tris-HCl (pH = 7.8) at 37 °C in the darkness for 1 h. Secondly, the incubated mixture solutions were transferred to 10 KDa ultrafiltration membranes that were immersed in 500 µL incubation buffer before using, and then centrifuged at 10,000 rpm for 10 min at 25 °C. Thirdly, the remaining mixture solutions were washed with 300 µL of Tris-HCl solution (pH = 7.8) and centrifuged at 8,000 rpm for 5 min at 25 °C for 3 times to remove the potential free or non-specific bound components. Next, 300 µL of 90% (*v*/*v*) MeOH-H_2_O was added and incubated for 10 min, and the mixed solutions were centrifuged at 10,000 rpm for another 10 min at 25 °C for 3 times to release those components with specific bindings to enzymes from the enzyme-ligand complexes. Finally, those filtrates were collected, freeze-dried and reconstituted in 50 µL MeOH immediately prior to HPLC-UV/ESI-MS/MS analysis.

#### 3.5.2. HPLC-UV/ESI-MS/MS Analysis

Thermo Accela 600 series HPLC tandem TSQ Quantum Access MAX mass spectrometer (Thermo Fisher Scientific, San Jose, CA, USA) was used to identify the components in WE-P and the ultrafiltration residues [40]. Briefly, a Waters Symmetry RP-C18 column (4.6 × 250 mm, 5 µm) was used for chromatographic analysis. The mobile phase consisted of 0.1% FA-H_2_O (A) and ACN (B). The optimized gradient HPLC elution procedures were set as follows: 0–28 min, 30% B; 28–32 min, 30–40% B, 32–60 min: 40–45% B. The injection volume was 10 µL, the flow rate was 0.8 mL/min, the column temperature was 30 °C, and the HPLC-UV chromatograms were obtained at a detection wavelength of 230 nm. The ESI-MS/MS parameters were applied as follows: the negative ion mode was set in this system, the spray voltage was set as 3.0 kV, the cone voltage was set as 40 V, the collision energy was set as 10 V according to the results of fragments; the sheath gas (N_2_) pressure was set as 40 psi, the aux gas pressure was set as 10 psi, the nebulizing gas flow rate was set as 6.0 L/min, the vaporizer temperature was set as 350 °C, the capillary temperature was set as 250 °C, the mass range from 50 to 1100 (*m*/*z*) was set in the full-scan mode. Finally, data acquisition and analysis were carried out by the Thermo Xcalibur ChemStation (Thermo Fisher Scientific, San Jose, CA, USA).

### 3.6. In Vitro COX-2 Inhibitory Assay

COX-2 (human) inhibitor screening assay kits were used to perform COX-2 inhibition assay to evaluate the COX-2 inhibition activity of compounds and verify the results of AUF-HPLC-MS/MS according to the manufacturer’s instructions [26,32]. Firstly, different concentration gradients solutions of samples were appropriately prepared with DMSO for later use. COX-2 cofactor working solution, COX-2 substrate, COX-2 working solution and COX-2 probe were prepared according to manufacturer’s instructions, and then diluted them with COX-2 assay buffer till their concentrations become one tenth of the original concentrations, respectively. Secondly, 150 µL Tris-HCl (pH = 7.8), 10 µL COX-2 cofactor working solution, 10 µL COX-2 working solution and 10 µL sample solution were successively added in the 96-well black plates, and then well-mixed and incubated for 10 min at 37 °C. In the blank control group, an equal volume of COX-2 assay buffer was used to substitute COX-2 working solution; an equal volume of DMSO was used to substitute sample. In the 100% enzyme activity control group, an equal volume of DMSO was used to substitute sample. Thirdly, 10 µL COX-2 probe was added into each well. Finally, 10 µL of COX-2 substrate was quickly added into each well in ice bath, and incubated at 37 °C in the darkness for 5 min, and followed by the fluorescence measurement. The excitation and emission wavelengths were 560 and 590 nm, respectively. Indomethacin was set as a positive control. The experiments were performed in triplicate. The COX-2 inhibitory activity was expressed as IC_50_ which was calculated by plotting the concentration-inhibition response curve. The inhibition of COX-2 was calculated according to the following formula:

COX-2 inhibitory activity (%) = (F_100%Enzyme_ − F_Sample_)/ (F_100%Enzyme_ − F_Blank_) × 100% (1)

COX-2 inhibitory activity (%) was plotted against the concentration of samples to acquire the IC_50_. F_100%Enzyme_, fluorescence of 100% enzyme control group; F_Sample_, fluorescence of sample group; F_Blank_, fluorescence of blank group.

### 3.7. Cell Culture and Cell Proliferative Assay

Human non-small cell lung adenocarcinoma cell line (A549) and human cervical cancer cell line (Hela) were obtained from the American Type Culture Collection (ATCC, Manassas, VA, USA) and maintained in Ham’s F-12K medium supplemented with 10% fetal bovine serum (FBS), 1% penicillin, and 1% streptomycin in a cell incubator under humidified conditions with 5% CO_2_ at 37 °C, and the procedures were slightly modified from previous method [40]. To evaluate the proliferative activity of A549 cell line and Hela cell line, the SRB assay was carried out according to the manufacturer’s instructions (Beyotime, Shanghai, China). Briefly, these two cell lines were seeded into 96-well plates at a density of 1.0 × 10^4^ per well. After 24 h incubation, the tested samples were dissolved in the DMSO and sequentially diluted with cell culture medium and added to each well at the specified concentrations. Cell viability was assessed by SRB 48 h later. Absorbances was measured by a Tecan microplate reader (Tecan Infinite M200 Pro, Tecan Group Ltd., Männedorf, Switzerland) at a wavelength of 540 nm. The experiments were performed in triplicate. The anti-proliferative activities were expressed as IC_50_. The cell growth inhibition rate was calculated as follows:

% cell inhibition = (A_c_ − A_s_)/A_c_ × 100% (2)

where A_c_ and A_s_ are the absorbances of control and samples, respectively. The data were expressed as mean ± standard deviation (SD) of three replicates.

### 3.8. Statistical Analysis

All the data were expressed as mean ± SD of triplicate independent measurements. Software used for statistical analysis mainly included SPSS 16.0 (SPSS Inc., Chicago, IL, USA), OriginPro 2021 (OriginLab Corporation, Northampton, MA, USA), and Graphpad Prism 5.0 (GraphPad Software Inc., San Diego, CA, USA). Structure analysis of compounds were implemented with Chemoffice 18.0 (CambridgeSoft Corp., Cambridge, MA, USA), and MestreNova (Mestrelab Research SL, San Diego, CA, USA).

## 4. Conclusions

*W. ugandensis* is a kind of evergreen tree with distinctive aromatic smell and important medicinal usages, belonging to the Canellaceae family, mainly distributed in Africa. Up to now, the potential bioactive constituents responsible for the anti-inflammatory and anti-proliferative activities in *W. ugandensis* and their underlying mechanisms remain need to be further explored. In this work, an affinity ultrafiltration screening combining four corresponding drug targets, such as COX-2, 5-LOX, Top I and Top II with HPLC-MS/MS was used and 2 potential ligands against COX-2, 5-LOX, Top I and Top II from *W. ugandensis* were quickly screened out and identified. Subsequently, compounds **1** and **2** were further revealed to exert inhibitory activities on COX-2 with the IC_50_ values of 4.405 ± 0.249 µM and 1.948 ± 0.381 µM, respectively. Furthermore, the anti-inflammatory activities of compounds **1** and **2** on COX-2 might be correlated to the highly binding capacities with COX-2 and 5-LOX (in Section 2.2.). In addition, compounds **1** and **2** also expressed anti-proliferative activities on Hela cell line with the IC_50_ values of 84.360 ± 1.794 µM and 72.803 ± 0.839 µM, respectively. It was also found that the anti-proliferative activities of compounds **1** and **2** were consistent with the results of affinity ultrafiltration screening (in Section 2.2.), which suggested that the potential anti-proliferative mechanisms of compounds **1** and **2** might be related to Top I and Top II. Furthermore, it also revealed for the first time that neolignanamides might be the potential bioactive components of *W. ugandensis* to exert anti-inflammatory and anti-proliferative activities. In summary, this study showcased an integrative strategy for the fast screening and subsequent identification of the potential bioactive components with anti-inflammatory and anti-proliferative activities from the crude extracts of a medicinal plant of interest combining correspondingly multiple target enzymes (COX-2, 5-LOX, Top I and Top II) with affinity ultrafiltration HPLC-MS/MS, and it would also provide a good guidance to search for effective agents with anti-inflammatory and anti-proliferative activities from medicinal plants of interest. 

## Figures and Tables

**Table 1:** The relative binding strength (RBS) values of representative peaks 1 and 2 from *W. ugandensis* in AUF-HPLC-MS/MS.

No.	Rt (min)	UF-RBS (%) ^#^			
		COX-2	5-LOX	Top I	Top II
**1**	28.64	20.15 ± 0.12	16.74 ± 0.65	34.25 ± 2.81	24.53 ± 1.54
**2**	36.67	27.39 ± 1.58	42.00 ± 1.26	76.2 ± 5.20	58.57 ± 3.82

^#^ Data were expressed as means ± standard deviation (n = 3); UF: Ultrafiltration; RBS: Relative binding strength; Rt: Retention time; COX-2: Cyclooxygenase-2; 5-LOX: 5-Lipoxygenase; Top I: Topoisomerase I; Top II: Topoisomerase II.

**Table 2:** The identification of corresponding peaks in Figure 2 in WE-P from *W. ugandensis* using HPLC-MS/MS and UPLC-QTOF-MS/MS.

No.	AUF-HPLC-MS/MS			UPLC-QTOF-MS/MS ^#^				Identified Compounds
	Rt (min)	Observed Mass *m*/*z* *	MS/MS Fragments	Observed Mass *m*/*z* *	Calculated Mass *m*/*z* * (Δ ppm)	Molecular Formula	MS/MS Fragments	
**1**	28.64	623	485 (4.30), 461 (73.00), 431 (11.40), 298 (100), 282 (16.85), 267 (8.37)	625.2546 **	625.2544 ** (0.32)	C_36_H_36_N_2_O_8_	488 (1.20), 462 (20.71), 351 (51.09), 325 (100), 307 (31.23), 293 (11.37), 201 (1.71), 138 (2.64), 121 (7.31)	*N*-*cis*-grossamide ^a^
**2**	36.67	623	622 (2.40), 471 (12.43), 460 (100), 445 (18.72), 432 (32.44), 416 (7.55), 401 (4.32), 351 (5.81), 307 (4.40), 297 (79.60), 282 (10.74)	625.2548 **	625.2544 ** (0.64)	C_36_H_36_N_2_O_8_	625 (35.87), 488 (10.84), 462 (93.45), 351 (42.94), 325 (100), 307 (9.98), 293 (2.27), 201 (1.58), 121 (17.01)	*N*-*trans*-grossamide ^a^
**3**	42.22	263	263 (50.10), 235 (22.46), 219 (100), 201 (33.88), 183 (24.12), 173 (8.38), 146 (3.73), 97 (1.53), 71 (12.36)	263.1298	263.1289 (3.42)	C_15_H_20_O_4_	263 (100), 235 (1.47), 219 (7.93), 191 (1.49)	7-hydroxywinterin ^b^
**4**	43.81	265	265 (57.24), 237 (9.03), 221 (100), 203 (4.06), 185 (8.93), 175 (0.20), 149 (0.11), 97 (0.25), 71 (10.76)	265.1445	265.1445 (0.00)	C_15_H_22_O_4_	265 (100), 237 (3.08), 221 (63.34), 203 (2.04), 185 (2.61), 71 (17.89)	ugandenial A ^a^
**5**	46.23	323	323 (11.88), 280 (4.56), 264 (100), 235 (15.16), 219 (14.69), 191 (7.94), 175 (1.85), 147 (2.63), 59 (4.67)	323.1508	323.1500 (2.48)	C_17_H_24_O_6_	323 (94.57), 279 (7.18), 263 (100), 235 (27.05), 219 (15.90), 201 (4.96), 191 (2.15), 147 (3.86), 59 (31.03)	11*α*-hydroxycinnamosmolide ^a^
**6**	47.65	237	237 (100), 219 (4.34), 193 (11.14), 163 (1.65), 145 (0.09), 106 (0.09)	237.1491	237.1496 (−2.11)	C_14_H_22_O_3_	237 (25.19), 191 (100), 177 (8.02), 163 (29.86), 147 (8.31), 135 (1.69), 107 (3.23)	polygonal acid ^a^
**7**	49.30	391	391 (51.79), 347 (85.39), 328 (13.48), 275 (68.95), 257 (60.29), 229 (100), 185 (0.72), 115 (25.78)	-	-	-	-	unknown

^#^ Data were tested from isolated compounds; *: quasi-molecular ion showed as [M-H]^−^; **: quasi-molecular ion showed as [M+H]^+^; ^a^: Identified according to published literatures and compared with the isolated compounds; ^b^: New compounds; Rt: Retention time; AUF-HPLC-MS/MS: Combination of affinity ultrafiltration and high-performance liquid chromatography-mass spectrometry; UPLC-QTOF-MS/MS: Ultra high performance liquid chromatography coupled to quadrupole time flight spectrometry; -: Not tested; unknown: Not identified compounds.

**Table 3:** ^1^H-NMR and ^13^C-NMR data for compounds **3** in methanol-*d*_4_.

Position	Compound 3 ^a^	
	*δ*_H_	*δ*_C_
1	1.32 (1H, overlap), 2.51 (1H, dt, *J* = 13.0, 3.6 Hz)	35.8 (t)
2	1.56 (1H, dq-like, *J* = 13.6, 3.6 Hz) 1.78 (1H, dt, *J* = 13.6, 3.6 Hz)	19.4 (t)
3	1.31 (1H, overlap), 1.52 (1H, m)	42.7 (t)
4	-	33.8 (s)
5	1.65 (1H, d, *J* = 13.0 Hz)	47.0 (d)
6	1.72 (1H, dd-like, *J* = 13.0, 4.1 Hz), 1.92 (1H, d-like, *J* = 14.0 Hz)	29.4 (t)
7	4.53 (1H, d-like, *J* = 3.0 Hz)	60.3 (d)
8	-	139.8 (s)
9	-	153.9 (s)
10	-	38.1 (s)
11	-	172.7 (s)
12	-	172.3 (s)
13	0.96 (3H, s)	33.6 (q)
14	0.94 (3H, s)	21.9 (q)
15	1.18 (3H, s)	19.1 (q)

^a^: 600 MHz for ^1^H NMR and 150 MHz for ^13^C NMR. -: No signal.

## Data Availability

Not applicable.

## Supplementary Materials

The following are available at https://www.mdpi.com/article/10.3390/ph14040313/s1, Figure S1: ^1^H NMR (600 MHz) spectrum of compound **3** in Methanol-*d*_4_, Figure S2: ^13^C NMR and DEPT (150 MHz) spectrum of compound **3** in Methanol-*d*_4_, Figure S3: ^1^H-^1^H COSY (600 MHz) spectrum of compound **3** in Methanol-*d*_4_, Figure S4: HSQC (600 MHz) spectrum of compound **3** in Methanol-*d*_4_, Figure S5: HMBC (600 MHz) spectrum of compound **3** in Methanol-*d*_4_, Figure S6: ^1^H NMR (600 MHz) spectrum of compound **1** in Methanol-*d*_4_, Figure S7: ^13^C NMR (150 MHz) spectrum of compound **1** in Methanol-*d*_4_, Figure S8: ^1^H NMR (600 MHz) spectrum of compound **2** in Methanol-*d*_4_, Figure S9: ^13^C NMR (150 MHz) spectrum of compound **2** in Methanol-*d*_4_, Figure S10: ^1^H NMR (600 MHz) spectrum of compound **4** in Methanol-*d*_4_, Figure S11: ^13^C NMR (150 MHz) spectrum of compound **4** in Methanol-*d*_4_, Figure S12: ^1^H NMR (600 MHz) spectrum of compound **5** in Methanol-*d*_4_, Figure S13**:** ^13^C NMR (150 MHz) spectrum of compound **5** in Methanol-*d*_4_, Figure S14: ^1^H NMR (600 MHz) spectrum of compound **6** in Methanol-*d*_4_, Figure S15: ^13^C NMR (150 MHz) spectrum of compound **6** in Methanol-*d*_4_.

## References

[1] Leonard C.M., Viljoen A.M. (2015). *Warburgia*: A comprehensive review of the botany, traditional uses and phytochemistry. J. Ethnopharmacol..

[2] Wube A.A., Gibbons S., Asres K., Streit B., Adams M., Bauer R., Bucar F. (2006). In vitro 12(*S*)-HETE and leukotriene metabolism inhibitory activity of sesquiterpenes of *Warburgia ugandensis*. Planta. Med..

[3] Xu M., Litaudon M., Krief S., Martin M.T., Kasenene J., Kiremire B., Dumontet V., Guéritte F. (2009). Ugandenial A, a new drimane-type sesquiterpenoid from *Warburgia ugandensis*. Molecules.

[4] Mbieda J.N., Lissouck D., Onguene P.P.A., Amana B.A., Ongagna J.M., Toze F.A., Mama D.B. (2021). Insight into the antioxidant and antiradical properties of colorotane sesquiterpenes extracted from *Warburgia ugandensis*: Theoretical evaluation. Struct. Chem..

[5] Mbwambo Z.H., Erasto P., Innocent E., Masimba P.J. (2009). Antimicrobial and cytotoxic activities of fresh leaf extracts of *Warburgia ugandensis*. Tanzania. J. Health Res..

[6] Merawie Y., Sahile S., Moges F., Husen A. (2013). Antimicrobial activity of crude and semi-purified fractions of *Warburgia ugandensis* against some pathogens. J. Coast. Life Med..

[7] Drage S., Mitter B., Engelmeier D., Chobot V., Gorfer M., Muchugi A., Jamnadass R.H., Sessitsch A., Hadacek F. (2017). Antimicrobial drimane sesquiterpenes contribute to balanced antagonism but do not structure bacterial and fungal endophytes in the African pepper bark tree *Warburgia ugandensis*. Front. Ecol. Evol..

[8] Drage S., Mitter B., Tröls C., Muchugi A., Jamnadass R.H., Sessitsch A., Hadacek F. (2014). Antimicrobial drimane sesquiterpenes and their effect on endophyte communities in the medical tree *Warburgia ugandensis*. Front. Microbiol..

[9] Kubo I., Fujita K., Lee S.H., Ha T.J. (2005). Antibacterial activity of polygodial. Phytother. Res..

[10] Ngure P.K., Tonui W.K., Ingonga J., Mutai C., Kigondu E., Ng’ang’a Z., Rukunga G., Kimutai A. (2009). In vitro antileishmanial activity of extracts of *Warburgia ugandensis* (Canellaceae), a Kenyan medicinal plant. J. Med. Plants Res..

[11] Irungu B.N., Rukunga G.M., Mungai G.M., Muthaura C.N. (2007). In vitro antiplasmodial and cytotoxicity activities of 14 medicinal plants from Kenya. S. Afr. J. Bot..

[12] Lacroix D., Prado S., Kamoga D., Kasenene J., Namukobe J., Krief S., Dumontet V., Mouray E., Bodo B., Brunois F. (2011). Antiplasmodial and cytotoxic activities of medicinal plants traditionally used in the village of Kiohima, Uganda. J. Ethnopharmacol..

[13] Coussens L.M., Werb Z. (2002). Inflammation and cancer. Nature.

[14] Arbyn M., Weiderpass E., Bruni L., de Sanjosé S., Saraiya M., Ferlay J., Bray F. (2020). Estimates of incidence and mortality of cervical cancer in 2018: A worldwide analysis. Lancet. Glob. Health.

[15] Bray F., Ferlay J., Soerjomataram I., Siegel R.L., Torre L.A., Jemal A. (2018). Global cancer statistics 2018: GLOBOCAN estimates of incidence and mortality worldwide for 36 cancers in 185 countries. CA Cancer. J. Clin..

[16] Crusz S.M., Balkwill F.R. (2015). Inflammation and cancer: Advances and new agents. Nat. Rev. Clin. Oncol..

[17] Banik K., Ranaware A.M., Harsha C., Nitesh T., Girisa S., Deshpande V., Fan L., Nalawade S.P., Sethi G., Kunnumakkara A.B. (2020). Piceatannol: A natural stilbene for the prevention and treatment of cancer. Pharmacol. Res..

[18] Ashrafizadeh M., Zarrabi A., Hushmandi K., Hashemi F., Moghadam E.R., Owrang M., Hashemi F., Makvandi P., Goharrizi M.A.S.B., Najafi M. (2021). Lung cancer cells and their sensitivity/resistance to cisplatin chemotherapy: Role of microRNAs and upstream mediators. Cell. Signal..

[19] Baglini E., Salerno S., Barresi E., Robello M., Da Settimo F., Taliani S., Marini A.M. (2021). Multiple topoisomerase I (TopoI), topoisomerase II (TopoII) and tyrosyl-DNA phosphodiesterase (TDP) inhibitors in the development of anticancer drugs. Eur. J. Pharm. Sci..

[20] Mohammed H.H.H., Abbas S.H., Hayallah A.M., Abuo-Rahma G.E.D.A., Mostafa Y.A. (2021). Novel urea linked ciprofloxacin-chalcone hybrids having antiproliferative topoisomerases I/II inhibitory activities and caspases-mediated apoptosis. Bioorg. Chem..

[21] Chang J.Y., Guo X., Chen H.X., Jiang Z.L., Fu Q., Wang H.K., Bastow K.F., Zhu X.K., Guan J., Lee K.H. (2000). Unique biochemical, cytotoxic, and antitumor activity of camptothecin and 4*β*-amino-4′-*O*-demethylepipodophyllotoxin conjugates. Biochem. Pharmacol..

[22] Cragg G.M., Newman D.J. (2005). Plants as a source of anti-cancer agents. J. Ethnopharmacol..

[23] Pommier Y., Leo E., Zhang H.L., Marchand C. (2010). DNA topoisomerases and their poisoning by anticancer and antibacterial drugs. Chem. Biol..

[24] Rolle C.E., Kanteti R., Surati M., Nandi S., Dhanasingh I., Yala S., Tretiakova M., Arif Q., Hembrough T., Brand T.M. (2014). Combined MET inhibition and topoisomerase I inhibition block cell growth of small cell lung cancer. Mol. Cancer Ther..

[25] Singh S., Pandey V.P., Yadav K., Yadav A., Dwivedi U.N. (2020). Natural products as anti-cancerous therapeutic molecules targeted towards topoisomerases. Curr. Protein Pept. Sci..

[26] Xie L.L., Lee D.Y.W., Shang Y., Cao X.T., Wang S.Q., Liao J., Zhang T., Dai R.H. (2020). Characterization of spirostanol glycosides and furostanol glycosides from *Anemarrhenae rhizoma* as dual targeted inhibitors of 5-lipoxygenase and cyclooxygenase-2 by employing a combination of affinity ultrafiltration and HPLC/MS. Phytomedicine.

[27] Tiwari M. (2017). The role of serratiopeptidase in the resolution of inflammation. Asian J. Pharm. Sci..

[28] Ouellet M., Riendeau D., Percival M.D. (2001). A high level of cyclooxygenase-2 inhibitor selectivity is associated with a reduced interference of platelet cyclooxygenase-1 inactivation by aspirin. Proc. Natl. Acad. Sci. USA.

[29] Cho H., Yun C.W., Park W.K., Kong J.Y., Kim K.S., Park Y., Lee S., Kim B.K. (2004). Modulation of the activity of pro-inflammatory enzymes, COX-2 and iNOS, by chrysin derivatives. Pharmacol. Res..

[30] Rajaram A., Vanaja G.R., Vyakaranam P., Rachamallu A., Reddy G.V., Anilkumar K., Arunasree K.M., Dhyani A., Prasad N.K., Sharma S. (2018). Anti-inflammatory profile of *Aegle marmelos* (L) Correa (Bilva) with special reference to young roots grown in different parts of India. J. Ayurveda Integr. Med..

[31] Zhao A.Q., Li L., Li B., Zheng M.Z., Tsao R. (2016). Ultrafiltration LC-ESI-MS^n^ screening of 5-lipoxygenase inhibitors from selected Chinese medicinal herbs *Saposhnikovia divaricata*, *Smilax glabra*, *Pueraria lobata* and *Carthamus tinctorius*. J. Funct. Foods.

[32] Zhu H.B., Liu S., Li X., Song F.R., Liu Z.Q., Liu S.Y. (2013). Bioactivity fingerprint analysis of cyclooxygenase-2 ligands from *radix Aconiti* by ultrafiltration-UPLC-MS^n^. Anal. Bioanal. Chem..

[33] Petrovic N., Murray M. (2010). Using *N*,*N*,*N*′,*N*′-tetramethyl-*p*-phenylenediamine (TMPD) to assay cyclooxygenase activity in vitro. Methods Mol. Biol..

[34] Zimmermann K.C., Sarbia M., Weber A.A., Borchard F., Gabbert H.E., Schrör K. (1999). Cyclooxygenase-2 expression in human esophageal carcinoma. Cancer Res..

[35] El-Dash Y., Khalil N.A., Ahmed E.M., Hassan M.S.A. (2021). Synthesis and biological evaluation of new nicotinate derivatives as potential anti-inflammatory agents targeting COX-2 enzyme. Bioorg. Chem..

[36] Wube A.A., Bucar F., Gibbons S., Asres K. (2005). Sesquiterpenes from *Warburgia ugandensis* and their antimycobacterial activity. Phytochemistry.

[37] Maroyi A. (2014). The genus *Warburgia*: A review of its traditional uses and pharmacology. Pharm. Biol..

[38] Munigunti R., Mulabagal V., Calderón A.I. (2011). Screening of natural compounds for ligands to PfTrxR by ultrafiltration and LC-MS based binding assay. J. Pharm. Biomed. Anal..

[39] Tao Y., Cai H., Li W., Cai B. (2015). Ultrafiltration coupled with high-performance liquid chromatography and quadrupole-time-of-flight mass spectrometry for screening lipase binders from different extracts of *Dendrobium officinale*. Anal. Bioanal. Chem..

[40] Chen G.L., Wu J.L., Li N., Guo M.Q. (2018). Screening for anti-proliferative and anti-inflammatory components from *Rhamnus davurica* Pall. using bio-affinity ultrafiltration with multiple drug targets. Anal. Bioanal. Chem..

[41] Fratoni E., Claudino V.D., Yunes R.A., Franchi G.C., Nowill A.E., Filho V.C., Monache F.D., Malheiros A. (2016). Further drimane sesquiterpenes from *Drimys brasiliensis* stem barks with cytotoxic potential. Naunyn. Schmiedebergs. Arch. Pharmacol..

[42] King R.R., Calhoun L.A. (2005). Characterization of cross-linked hydroxycinnamic acid amides isolated from potato common scab lesions. Phytochemistry.

[43] Madikane V.E., Bhakta S., Russell A.J., Campbell W.E., Claridge T.D., Elisha B.G., Davies S.G., Smith P., Sim E. (2007). Inhibition of mycobacterial arylamine *N*-acetyltransferase contributes to anti-mycobacterial activity of *Warburgia salutaris*. Bioorg. Med. Chem..

[44] Sultana R., Hossain R., Adhikari A., Ali Z., Yousuf S., Choudhary M.I., Ali M.Y., Zaman M.S. (2011). Drimane-type sesquiterpenes from *Polygonum hydropiper*. Planta Med..

[45] Kioy D., Gray A.I., Waterman P.G. (1990). A comparative study of the stem-bark drimane sesquiterpenes and leaf volatile oils of *Warburgia ugandensis*, and *W. Stuhlmannii*. Phytochemistry.

[46] Appel H.H., Bond R.P.M., Overton K.H. (1963). Sesquiterpenoids-III the constitution and stereochemistry of valdiviolide, fuegin, winterin and futronolide. Tetrahedron.

[47] Fukuyama Y., Sato T., Miura I., Asakawa Y. (1985). Drimane-type sesqui- and norsesquiterpenoids from *Polygonum hydropiper*. Phytochemistry.

[48] Bastos J.K., Kaplan M.A.C., Gottlieb O.R. (1999). Drimane-type sesquiterpenoids as chemosystematic markers of Canellaceae. J. Braz. Chem. Soc..

[49] Rajab M.S., Ndegwa J.M. (2000). 11*α*-Hydroxy muzigadiolide, a novel drimane sesquiterpene from the stem bark of *warburgia ugandensis*. Bull. Chem. Soc. Ethiop..

[50] Mudyiwa M., Rajab M.S., Fronczek F.R., Watkins S.F. (2012). (1*R*,4*R*,5a*S*,7*S*,9a*S*)-7,9a-Dimethyl-6-methylene-3-oxo-1,3,4,5,5a,6,7,8,9,9a-decahydronaphtho-[1,2-c]furan-1,4-diyldiacetate. Acta Crystallogr. Sect. E Struct. Rep. Online.

[51] Opiyo S.A., Manguro L.O.A., Okinda-Owuor P., Ateka E.M., Lemmen P. (2011). 7*α*-Acetylugandensolide and antimicrobial properties of *Warburgia ugandensis* extracts and isolates against sweet potato pathogens. Phytochem. Lett..

[52] Yang B.Y., Yin X., Liu Y., Ye H.L., Zhang M.L., Guan W., Kuang H.X. (2019). Bioassay-guided isolation of lignanamides with potential anti-inflammatory effect from the roots of *Solanum melongena* L.. Phytochem. Lett..

[53] Nchiozem-Ngnitedem V.A., Omosa L.K., Bedane K.G., Derese S., Brieger L., Strohmann C., Spiteller M. (2020). Anti-inflammatory steroidal sapogenins and a conjugated chalcone-stilbene from *Dracaena usambarensis* Engl. Fitoterapia.

[54] Zhuang X.C., Chen G.L., Liu Y., Zhang Y.L., Guo M.Q. (2021). New lignanamides with antioxidant and anti-inflammatory activities screened out and identified from *Warburgia ugandensis* combining affinity ultrafiltration LC-MS with SOD and XOD enzymes. Antioxidants.

[55] Gao X.X., Gao Y.N., Wang D.D., Hu G.S., Yan T., Jia J.M., Wang A.H. (2021). Six novel lignanoids with complex structures from *Sigesbeckia glabrescens* Makino with their cytotoxic activities. Fitoterapia.

[56] Truong L.H., Cuong N.H., Dang T.H., Hanh N.T.M., Thi V.L., Hong H.T.T., Quang T.H., Nguyen H.D., Xuan C.N., Hoai N.N. (2020). Cytotoxic constituents from *Isotrema tadungense*. J. Asian Nat. Prod. Res..

[57] Ma C.Y., Liu W.K., Che C.T. (2002). Lignanamides and nonalkaloids components of *Hyoscyamus niger* seeds. J. Nat. Prod..

[58] Cardullo N., Pulvirenti L., Spatafora C., Musso N., Barresi V., Condorelli D.F., Tringali C. (2016). Dihydrobenzofuran neolignanamides: Laccase-mediated biomimetic synthesis and antiproliferative activity. J. Nat. Prod..

