# Supplementary material for: Identification of Anti-Inflammatory and Anti-Proliferative Neolignanamides from Warburgia ugandensis Employing Multi-Target Affinity Ultrafiltration and LC-MS
Source: Pharmaceuticals (Basel). 2021 Apr 1;14(4):313. doi: 10.3390/ph14040313 (PMC8065987; doi:10.3390/ph14040313)
Supplement: Supplementary file 1 [file pharmaceuticals-14-00313-s001.pdf]

pharmaceuticals

Supplementary Materials

Identification of Anti-inflammatory and Anti-proliferative Neolignanamides from *Warburgia ugandensis* Employing Multi-target Affinity Ultrafiltration and LC-MS

Xiao-Cui Zhuang [1,2,3,4,5], Yong-Li Zhang [1,3,4], Gui-Lin Chen [1,3,4], Ye Liu [1,3,4], Xiao-Lan Hu [6], Na Li [6], Jian-Lin Wu [6], and Ming-Quan Guo [1,3,4*]

[1] Key Laboratory of Plant Germplasm Enhancement and Specialty Agriculture, Wuhan Botanical Garden, Chinese Academy of Sciences, Wuhan 430074, China; zhuangxiaocui@yxnu.edu.cn (X. C. Z.); zhangyongli@wbgcas.cn (Y. L. Z.); liuye@wbgcas.cn (Y. L)
[2] University of Chinese Academy of Sciences, Beijing 100049, China;
[3] Sino-Africa Joint Research Center, Chinese Academy of Sciences, Wuhan 430074, China;
[4] Innovation Academy for Drug Discovery and Development, Chinese Academy of Sciences, Shanghai 201203, China;
[5] School of Chemical Biology and Environment, Yuxi Normal University, Yuxi 653100, China;
[6] State Key Laboratory of Quality Research in Chinese Medicine, Macau University of Science and Technology, Avenida Wai Long, Taipa, Macau SAR, China; 18098538ct30001@student.must.edu.mo (X. L. H.); nli@must.edu.mo (L. N.); jlwu@must.edu.mo (J. L. W.)
* Correspondence: glchen@wbgcas.cn; guomq@wbgcas.cn; Tel.: +86-027-87700850

Contents of Supporting Information

Figure S1. ^{1}H NMR (600 MHz) spectrum of compound **3** in Methanol-*d*4 2

Figure S2. ^{13}C NMR and DEPT (150 MHz) spectrum of compound **3** in Methanol-*d*4 2

Figure S3. ^{1}H-^{1}H COSY (600 MHz) spectrum of compound **3** in Methanol-*d*4 3

Figure S4. HSQC (600 MHz) spectrum of compound **3** in Methanol-*d*4 3

Figure S5. HMBC (600 MHz) spectrum of compound **3** in Methanol-*d*4 4

Figure S6. ^{1}H NMR (600 MHz) spectrum of compound **1** in Methanol-*d*4 5

Figure S7. ^{13}C NMR (150 MHz) spectrum of compound **1** in Methanol-*d*4 5

Figure S8. ^{1}H NMR (600 MHz) spectrum of compound **2** in Methanol-*d*4 6

Figure S9. ^{13}C NMR (150 MHz) spectrum of compound **2** in Methanol-*d*4 6

Figure S10. ^{1}H NMR (600 MHz) spectrum of compound **4** in Methanol-*d*4 7

Figure S11. ^{13}C NMR (150 MHz) spectrum of compound **4** in Methanol-*d*4 7

Figure S12. ^{1}H NMR (600 MHz) spectrum of compound **5** in Methanol-*d*4 8

Figure S13. ^{13}C NMR (150 MHz) spectrum of compound **5** in Methanol-*d*4 8

Figure S14. ^{1}H NMR (600 MHz) spectrum of compound **6** in Methanol-*d*4 9

Figure S15. ^{13}C NMR (150 MHz) spectrum of compound **6** in Methanol-*d*4 9

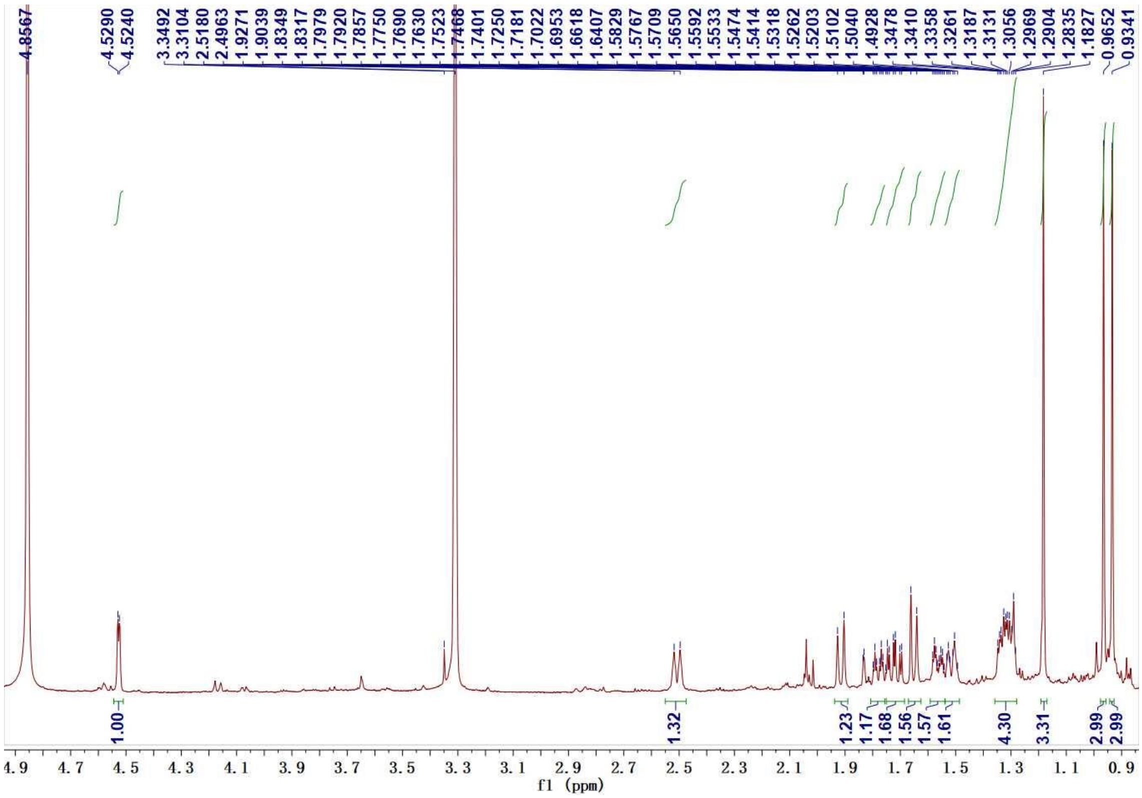

Figure S1. ^{1}H NMR (600 MHz) spectrum of compound **3** in Methanol-d_4

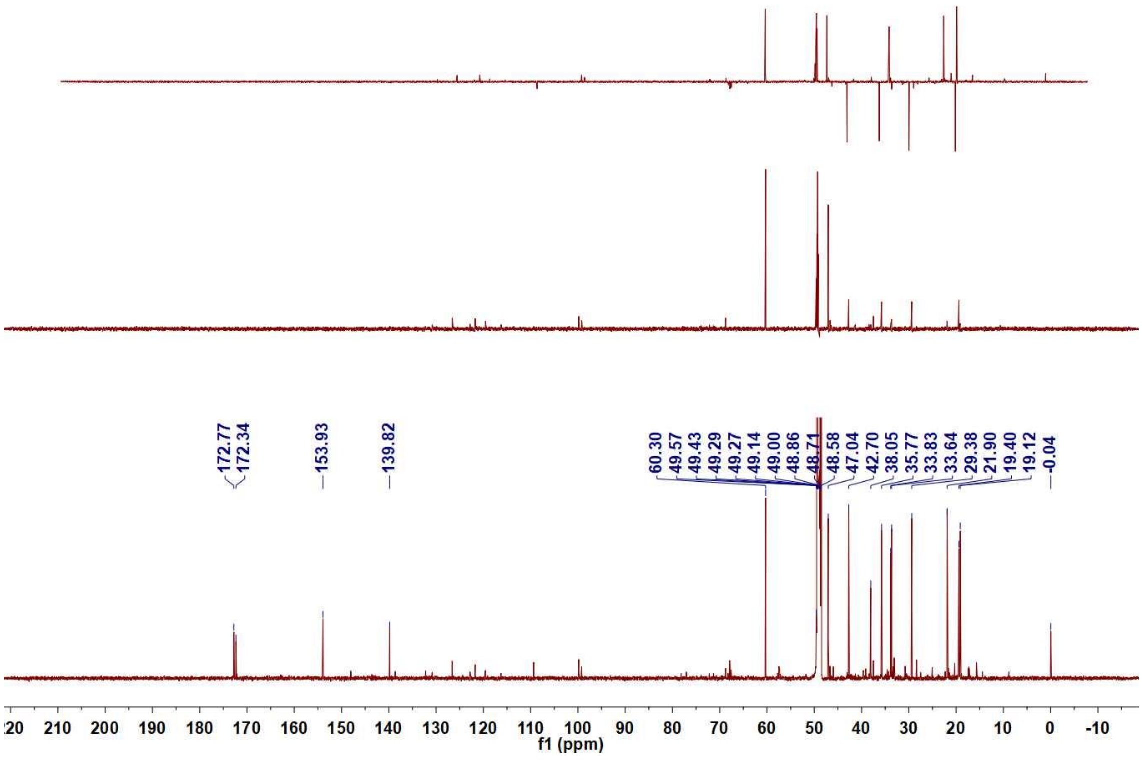

Figure S2. ^{13}C NMR and DEPT (150 MHz) spectrum of compound **3** in Methanol-d_4

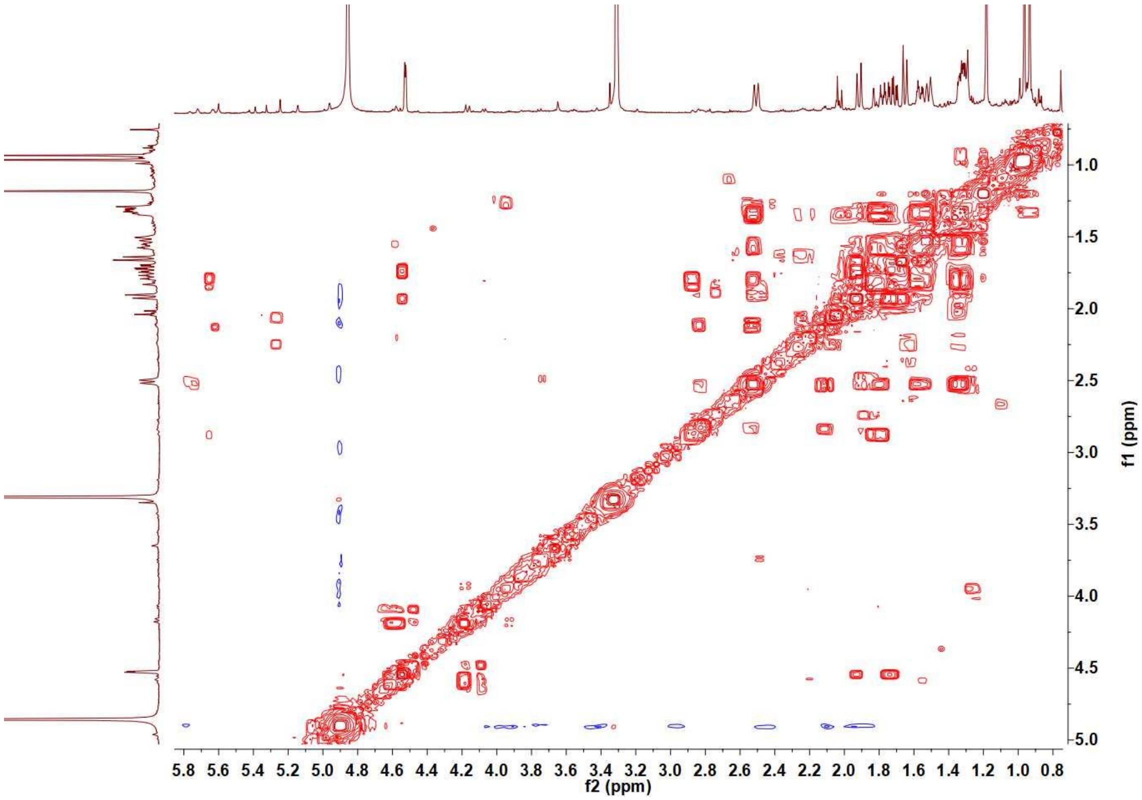

Figure S3. ^{1}H-^{1}H COSY (600 MHz) spectrum of compound **3** in Methanol-d_4

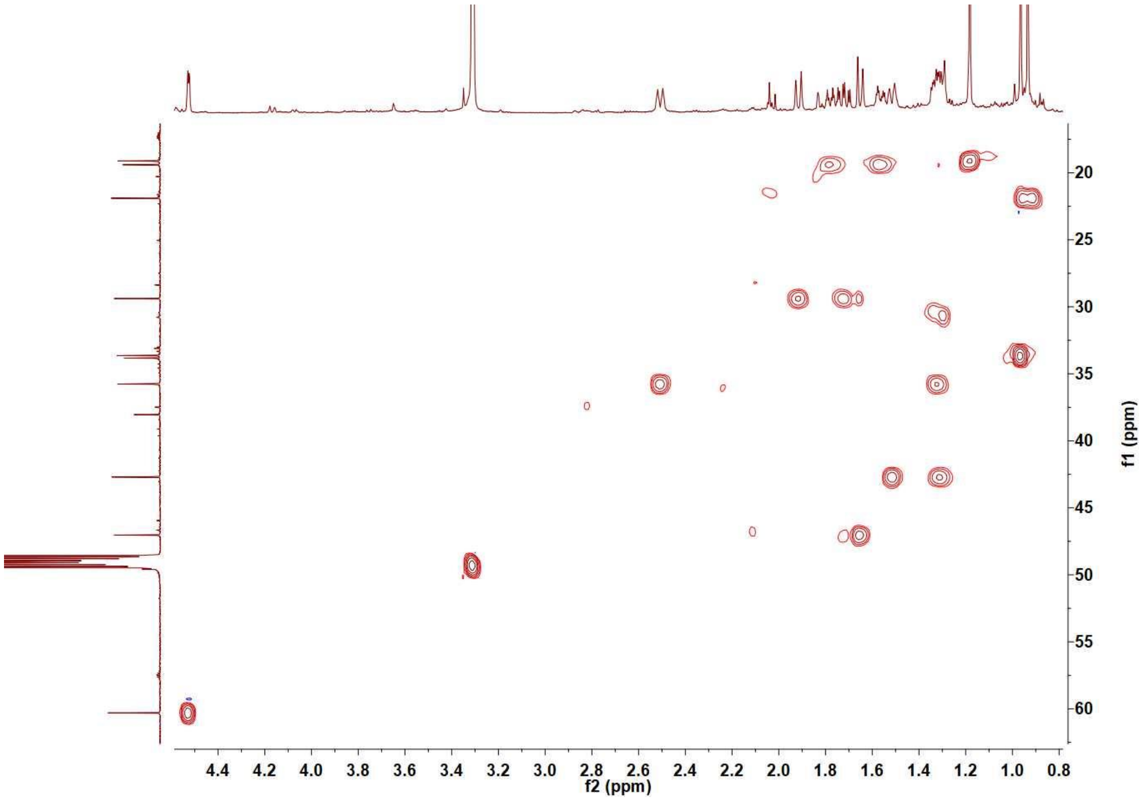

Figure S4. HSQC (600 MHz) spectrum of compound **3** in Methanol-d_4

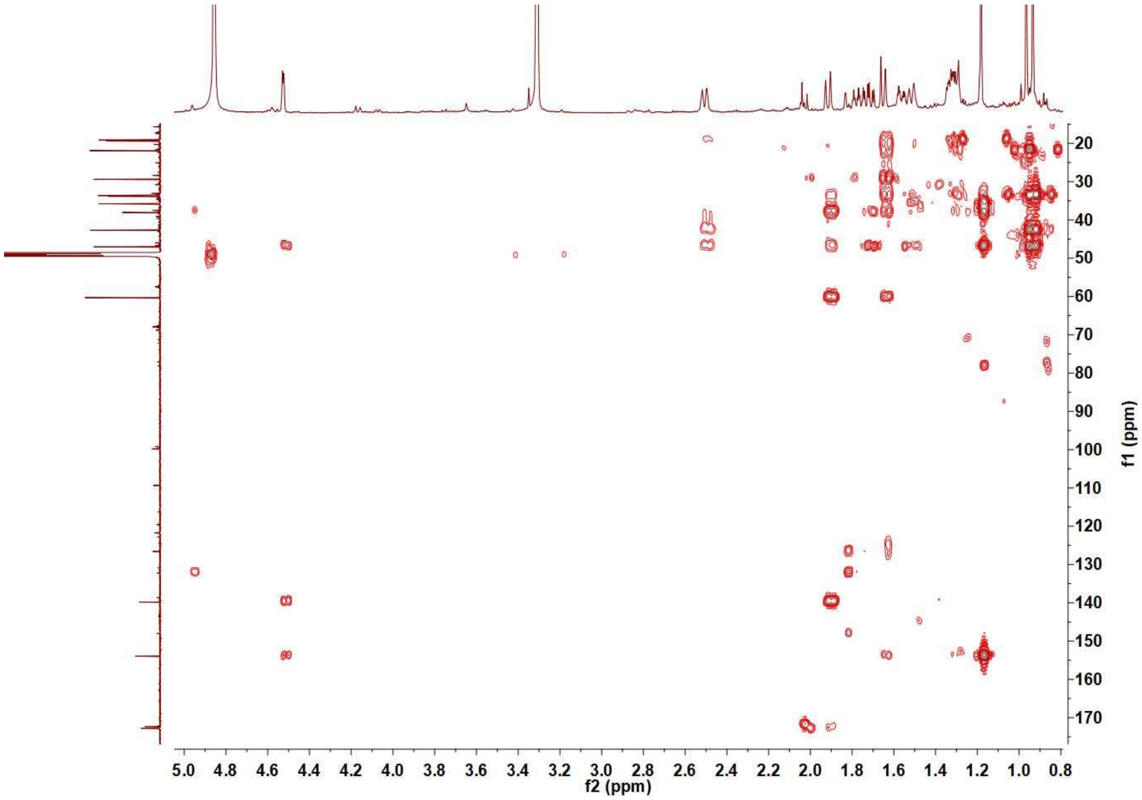

Figure S5. HMBC (600 MHz) spectrum of compound **3** in Methanol-d_4

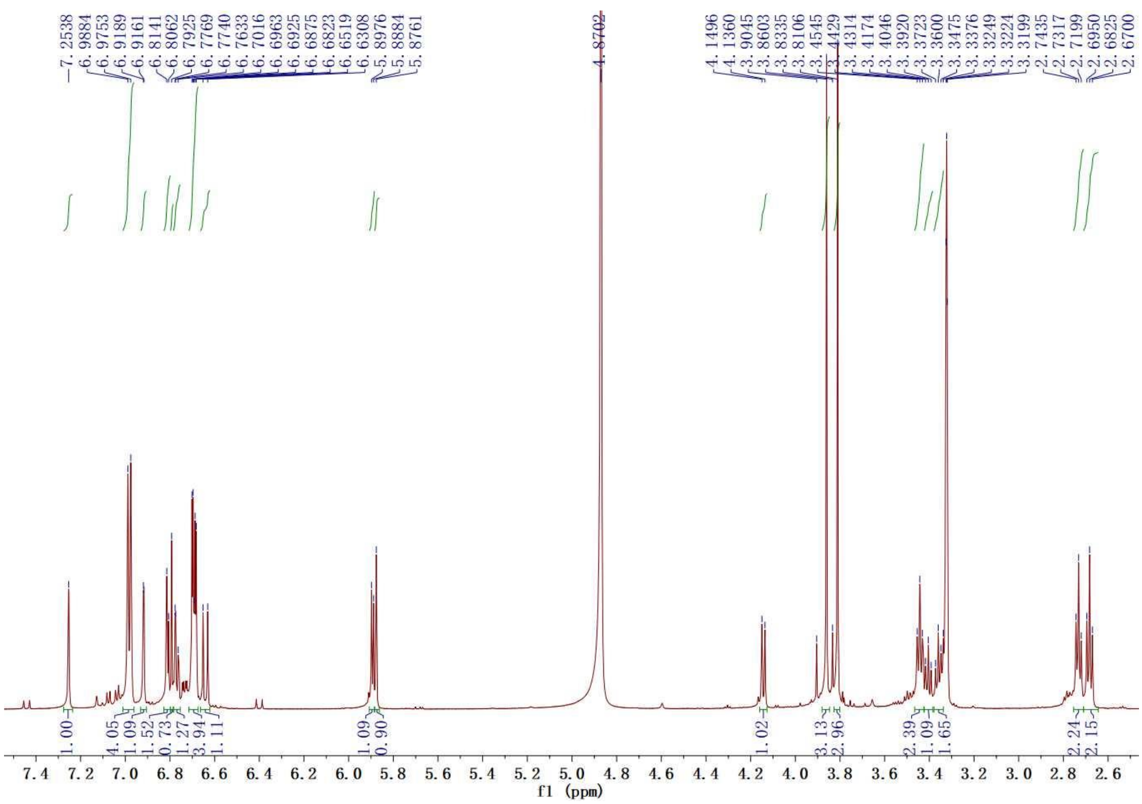

Figure S6. ^{1}H NMR (600 MHz) spectrum of compound **1** in Methanol-d_4

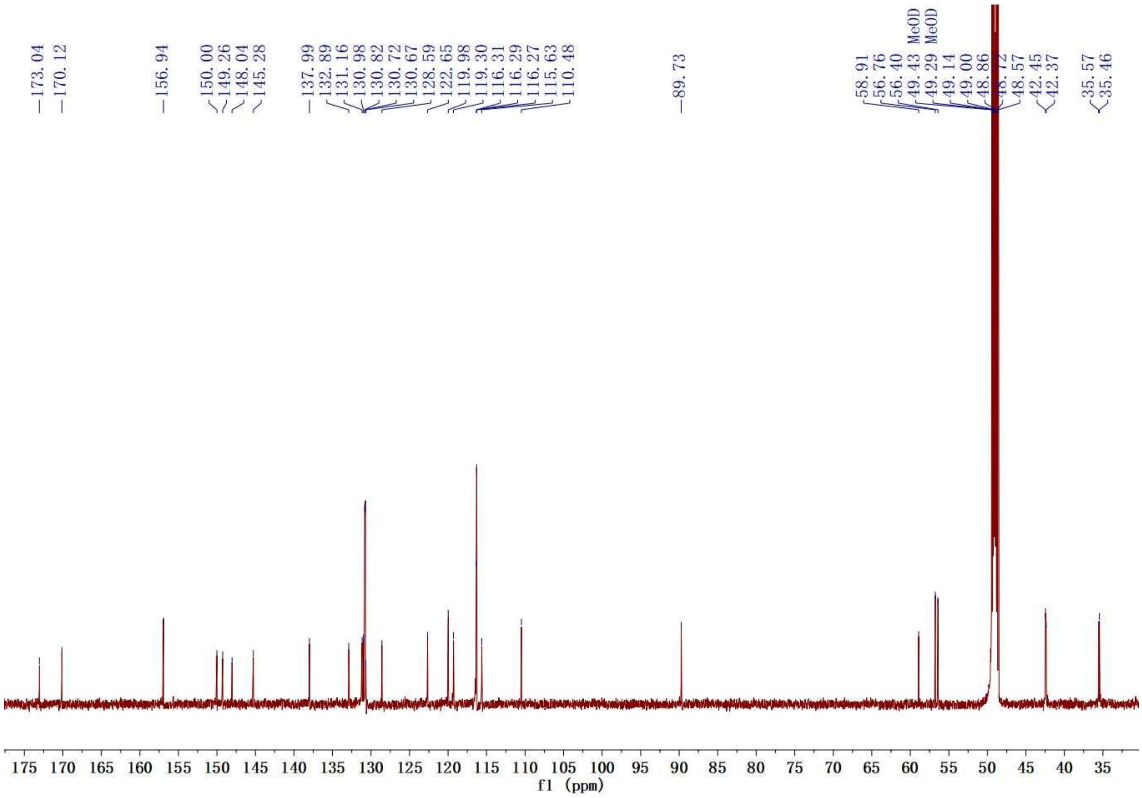

Figure S7. ^{13}C NMR (150 MHz) spectrum of compound **1** in Methanol-d_4

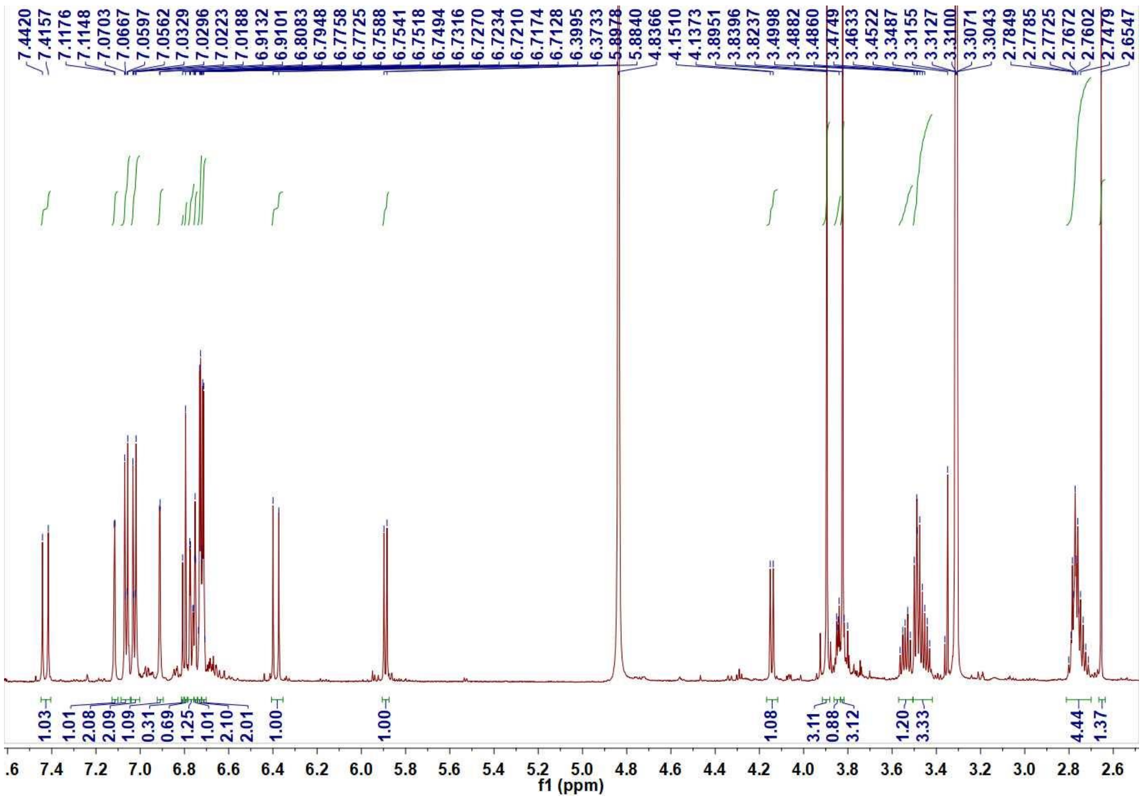

Figure S8. ^{1}H NMR (600 MHz) spectrum of compound **2** in Methanol-d_4

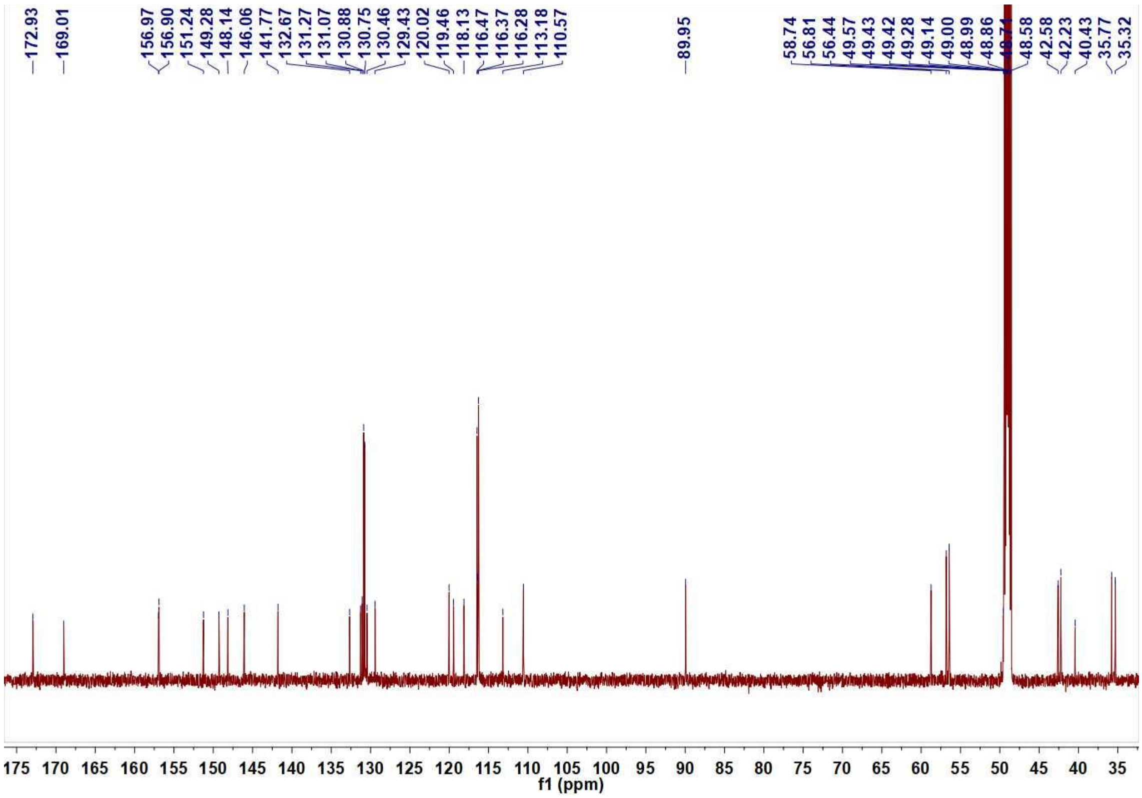

Figure S9. ^{13}C NMR (150 MHz) spectrum of compound **2** in Methanol-d_4

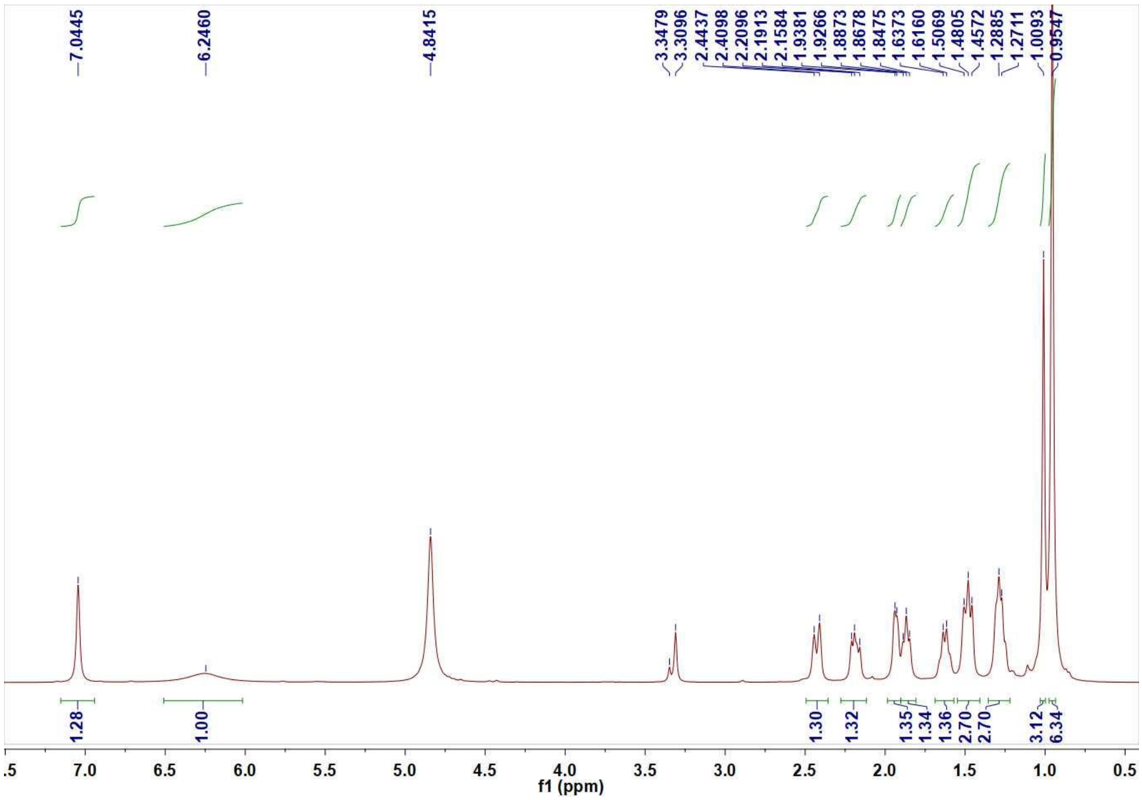

Figure S10. ^{1}H NMR (600 MHz) spectrum of compound **4** in Methanol-d_4

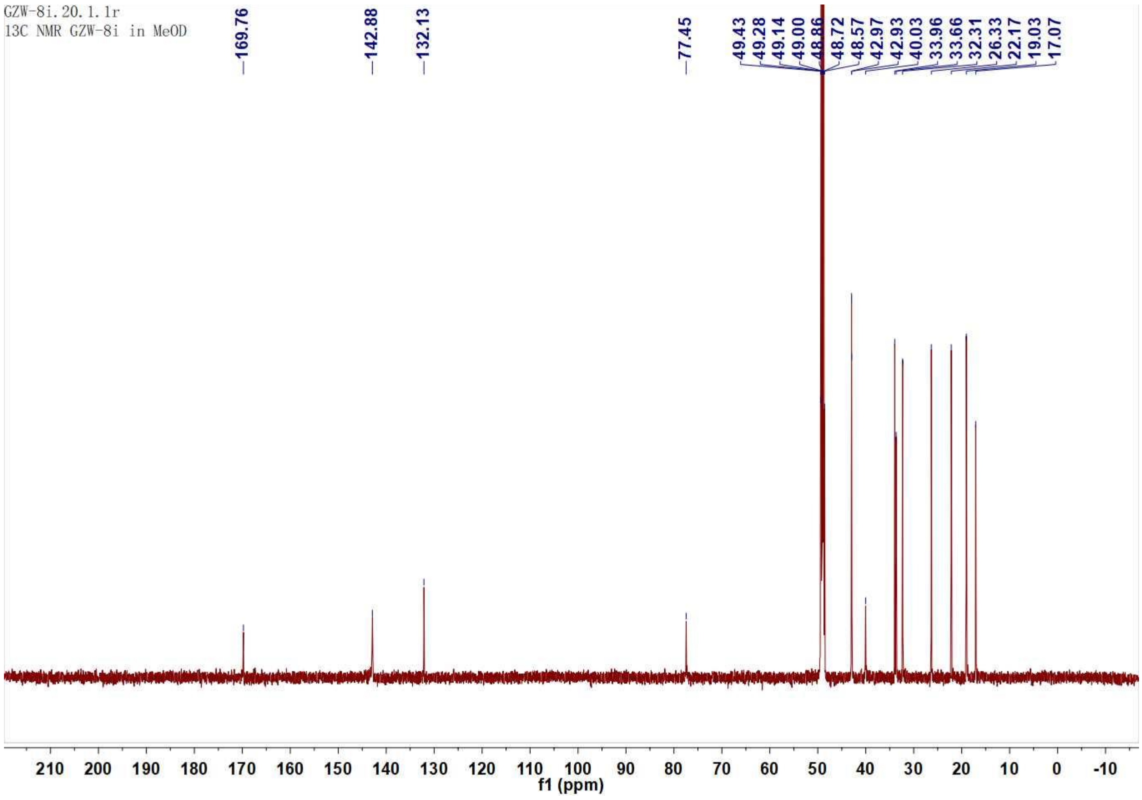

Figure S11. ^{13}C NMR (150 MHz) spectrum of compound **4** in Methanol-d_4

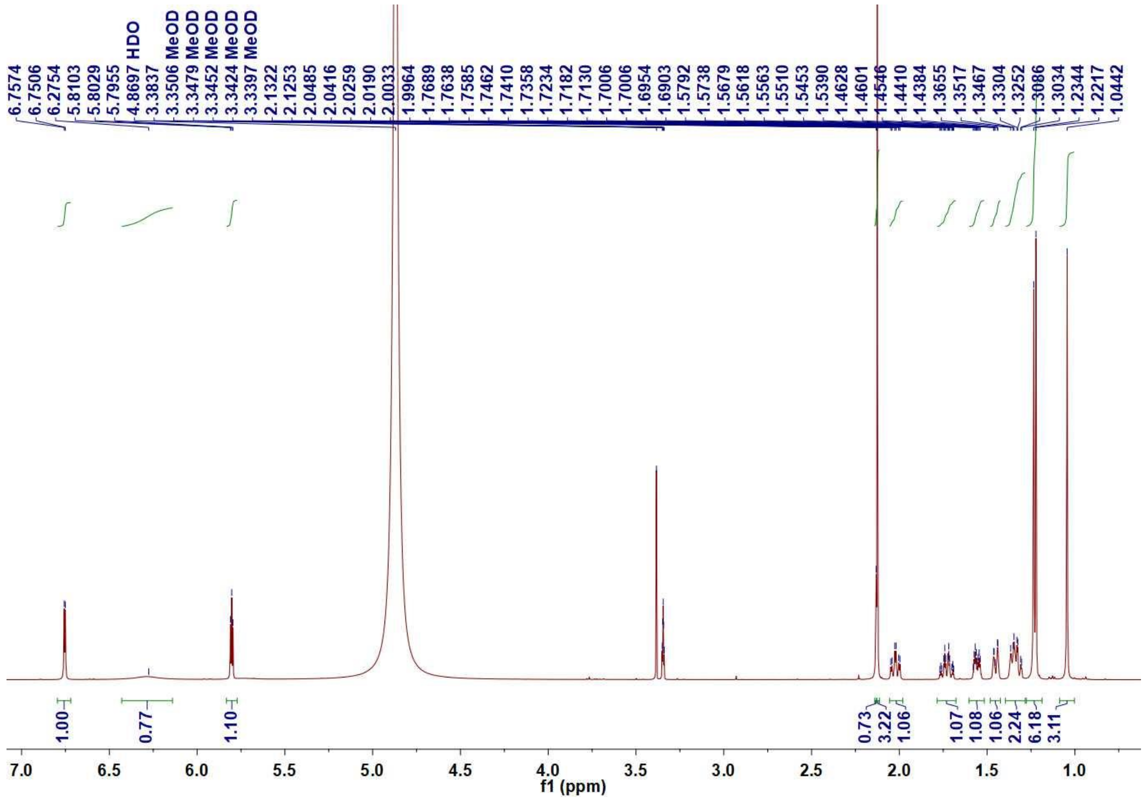

Figure S12. ^{1}H NMR (600 MHz) spectrum of compound **5** in Methanol-*d*4

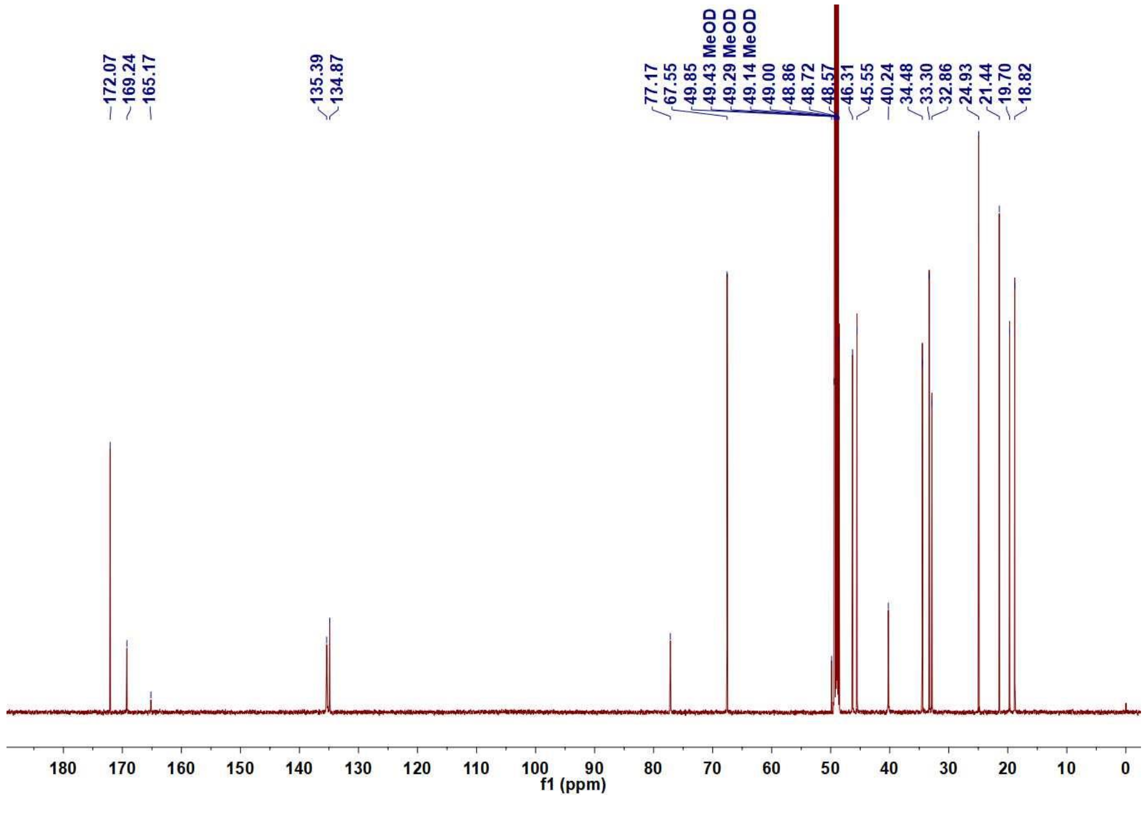

Figure S13. ^{13}C NMR (150 MHz) spectrum of compound **5** in Methanol-*d*4

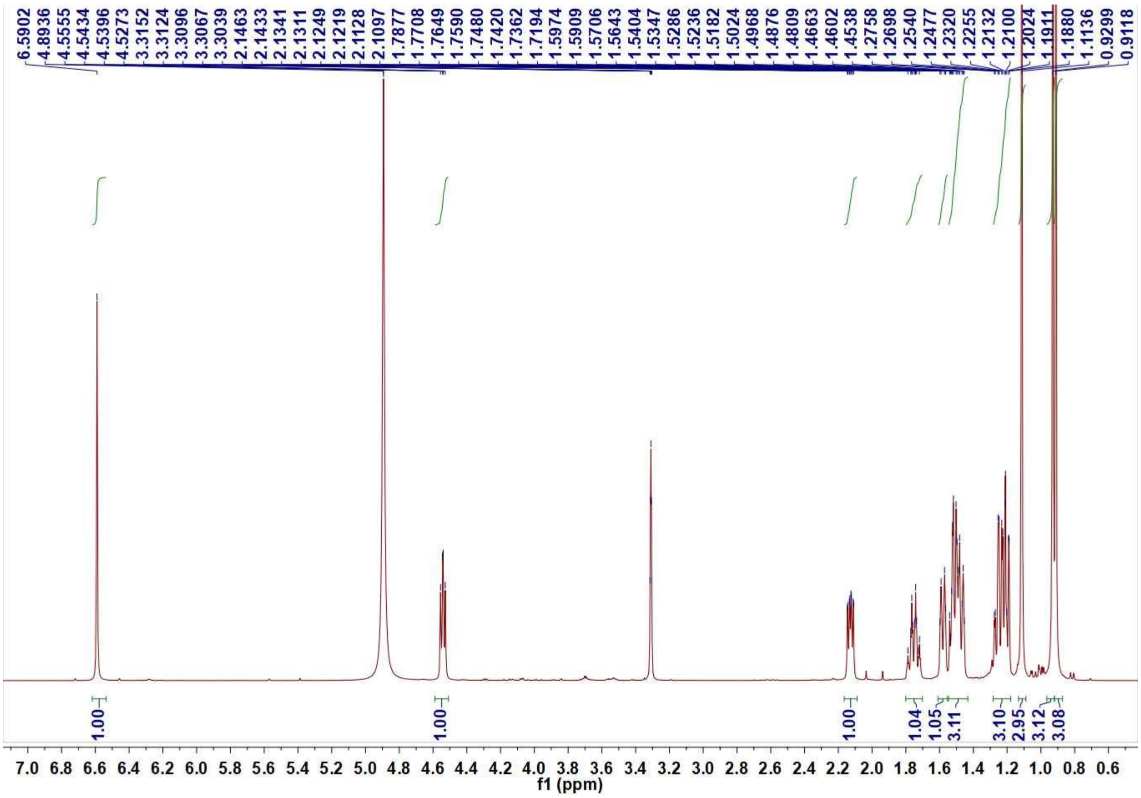

Figure S14. ^{1}H NMR (600 MHz) spectrum of compound **6** in Methanol-d_4

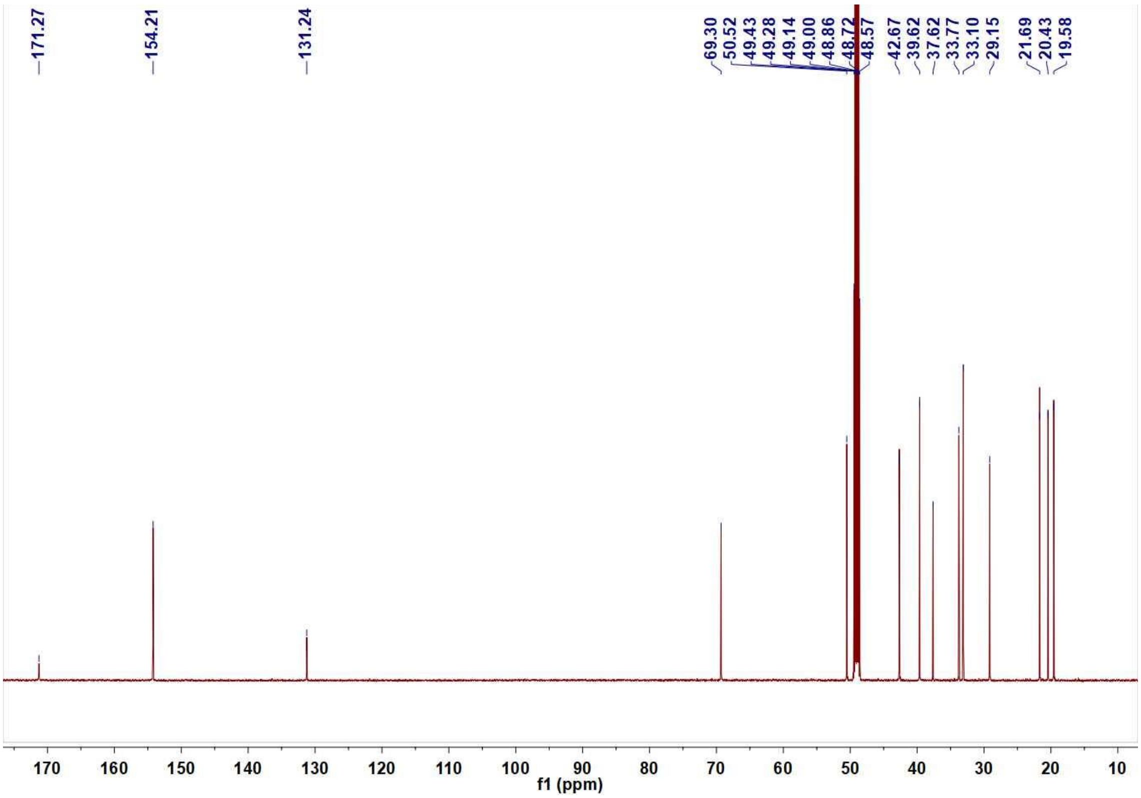

Figure S15. ^{13}C NMR (150 MHz) spectrum of compound **6** in Methanol-d_4
